# Suppression of cell division-associated genes by *Helicobacter pylori* attenuates proliferation of RAW264.7 monocytic macrophage cells

**DOI:** 10.1038/srep11046

**Published:** 2015-06-16

**Authors:** Grace Min Yi Tan, Chung Yeng Looi, Keith Conrad Fernandez, Jamuna Vadivelu, Mun Fai Loke, Won Fen Wong

**Affiliations:** 1 Department of Medical Microbiology; 2 Department of Pharmacology, Faculty of Medicine, University of Malaya, 50603 Kuala Lumpur, Malaysia; 3 Tropical Infectious Disease Research and Education Center, University of Malaya, 50603 Kuala Lumpur, Malaysia

## Abstract

*Helicobacter pylori* at multiplicity of infection (MOI ≥ 50) have been shown to cause apoptosis in RAW264.7 monocytic macrophage cells. Because chronic gastric infection by *H. pylori* results in the persistence of macrophages in the host’s gut, it is likely that *H. pylori* is present at low to moderate, rather than high numbers in the infected host. At present, the effect of low-MOI *H. pylori* infection on macrophage has not been fully elucidated. In this study, we investigated the genome-wide transcriptional regulation of *H. pylori-*infected RAW264.7 cells at MOI 1, 5 and 10 in the absence of cellular apoptosis. Microarray data revealed up- and down-regulation of 1341 and 1591 genes, respectively. The expression of genes encoding for DNA replication and cell cycle-associated molecules, including *Aurora-B kinase (AurkB)* were down-regulated. Immunoblot analysis verified the decreased expression of AurkB and downstream phosphorylation of Cdk1 caused by *H. pylori* infection. Consistently, we observed that *H. pylori* infection inhibited cell proliferation and progression through the G1/S and G2/M checkpoints. In summary, we suggest that *H. pylori* disrupts expression of cell cycle-associated genes, thereby impeding proliferation of RAW264.7 cells, and such disruption may be an immunoevasive strategy utilized by *H. pylori*.

*Helicobacter pylori* (*H. pylori*) is a Gram-negative, microaerophilic bacterium that colonizes the human gastric and duodenal mucosal epithelium. It is a well-established causative agent of gastritis and duodenal ulcer, and is a significant risk factor of gastric adenocarcinoma^1^. *H. pylori* is often incompletely eradicated and is able to persist in host for life^2^.

Gastritis in the acute *H. pylori* infection is predominantly mediated by macrophages^3^. A transient depletion of macrophages during *H. pylori* infection reduces the gastric pathology in animal model^3^. Normal gastric mucosa in an adult is populated by small population of macrophage^4^. During *H. pylori* infection, surface and secreted proteins from *H. pylori* act as chemoattractant and induce circulating monocytes to infiltrate the gastric epithelium^5^,^6^, which subsequently differentiate to enlarge the macrophage population at the infection site. Besides, the *H. pylori*-induced gastric epithelial damage enables the bacteria to enter lamina propria and encounter macrophages^7^. Macrophages infiltration into gastric mucosa is detected in the *H. pylori*-infected patients^8^, which can function to capture the bacteria^7^. Both M1 and M2 macrophages are detected in gastric biopsy specimens isolated from *H. pylori*-infected patients, indicating the importance of macrophages in host defense against *H. pylori*^9^,^10^. Macrophages respond to *H. pylori* infection by increasing surface expression of CD80, CD86 and HLA-DR accompanied by elevated secretion of cytokines including IL-12p70 and IL-23 that stimulate T_H_1 and T_H_17 responses, respectively^9^.

To maintain persistent infection of the host, *H. pylori* develops various immune evasion strategies to resist elimination by the host immune system, one of which is through delaying the macrophage-mediated phagocytosis^11^,^12^. Besides, chronic exposure to *H. pylori* impairs antigen presentation by macrophages, thus inhibiting development of T_H_1 cells and IFN-γ secretion^13^. Several studies have reported that at high MOIs, *H. pylori* causes abrupt cell death of monocytes^14^ and macrophages through activation of Erk-15, arginase II-16,17, or mitochondrial-dependent^18^,^19^ pathways. *H. pylori* is also reported to initiate cell death through autophagic mechanism^20^. Despite these data showing *H. pylori* induces monocytes and macrophage cell death, *in vivo* examination of patient samples detected a large number of these cells at the infection site^9^,^10^. We therefore hypothesize that *H. pylori* is most likely present in the stomach at levels that are not sufficient to trigger apoptosis in host macrophages and may instead be protective, as *H. pylori* at low MOIs reduces apoptotic cell death in B lymphocytes^21^. The crosstalk of macrophages and *H. pylori* at low MOIs, which at present has not been fully described, is important for understanding the host defense against *H. pylori*, particularly during initial and chronic infection stages.

In this study, we performed microarray analysis to investigate genome-wide gene expression by RAW264.7 monocytic macrophages infected with *H. pylori* at MOI 10. Our report showed that *H. pylori* suppressed the expression of genes that encode for DNA synthesis and cell cycle-associated molecules that functionally translated to disrupted proliferation and cell cycle progression in these *H. pylori*-infected RAW264.7 cells.

## Results

### *H. pylori* at MOI 10 activates monocytic macrophages cells

**Figure 1:**
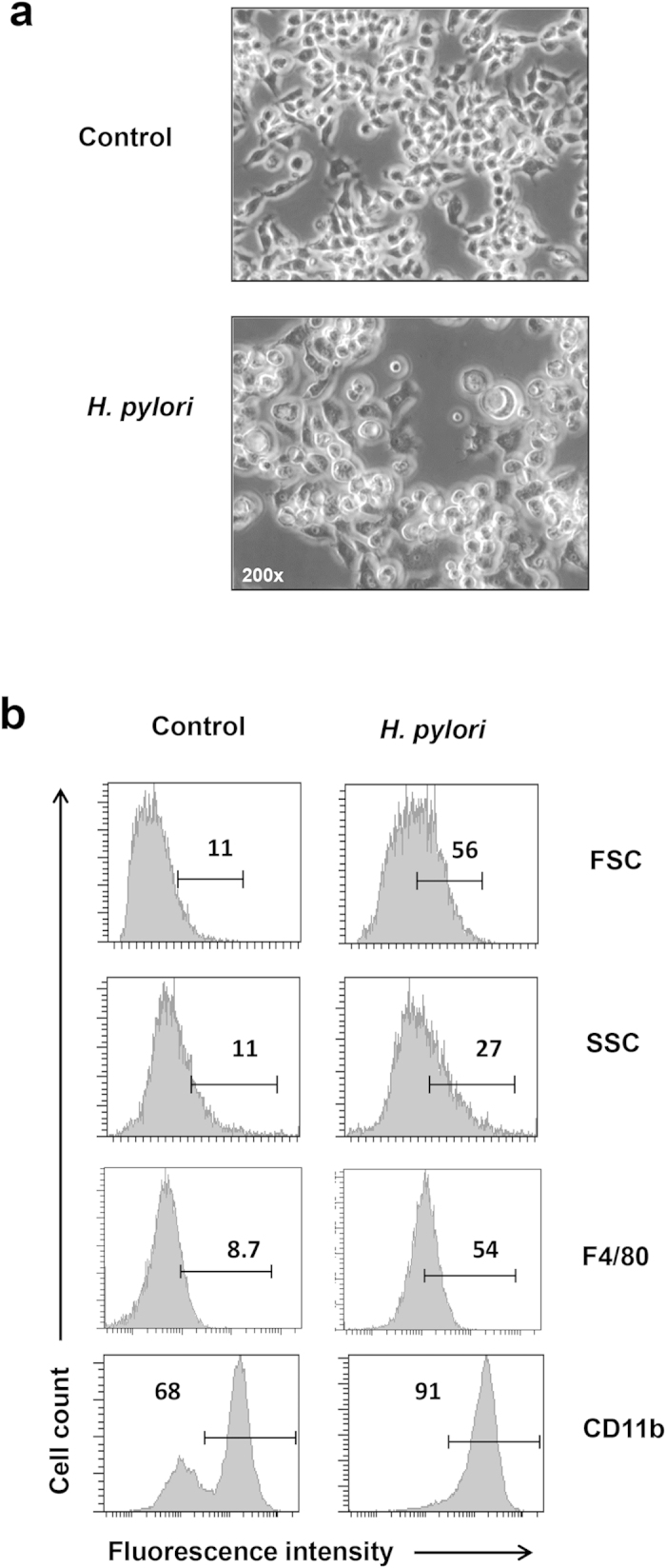
*H. pylori*-infected RAW264.7 monocytic macrophage cells. RAW264.7 cells were seeded at 5 × 10^5^ /ml and infected with the indicated MOIs of *H. pylori* for 24 h. (**a**) Representative pictures of control and infected cells viewed under light microscope. Objective 200×. (**b**) Flow cytometry analysis of the control and infected cells. Intensities of forward scatter (FCS) and side scatter (SSC) indicate the cell size and complexity, respectively. Numbers represent the percentages of cells in the gated area. Shown are representative data of three independent experiments.

To ascertain whether monocytic macrophages will be activated by *H. pylori*, we infected RAW264.7 cells with *H. pylori* Sydney strain 1 (SS1) at MOI 10. *H. pylori* SS1 is employed in this study as it is a well-established mouse-adapted pathogenic strain and its infectivity has been confirmed in RAW264.7 cells^16^. At 24 hours post infection (hpi), RAW264.7 cells were grossly enlarged (Fig. 1a), and increased intensities of forward scatter (FSC) and side scatter (SSC) parameters detected via flow cytometry verified the augmented cell size and complexity in the infected RAW264.7 cells (Fig. 1b). Besides, we observed that upon infection, RAW264.7 cells increased surface expression of macrophage markers F4/80 and CD11b, suggesting monocyte-to-macrophage differentiation. Uninfected controls were composed of undifferentiated monocytic macrophages displaying F4/80^low^ and CD11b (Mac-1)^medium/high^ phenotypes whereas infected cells exhibited F4/80^high^ and CD11b^high^ expression. Further, we observed no sign of apoptotic events within the infected macrophage population at MOI 1 to 10 (Supplementary Figure S1), providing support that *H. pylori* at these MOIs was capable of activating cells, but inadequate of inducing apoptotic cell death in RAW264.7 cells. On the contrary, at MOI of 100, *H. pylori* induced apoptosis (annexin^+^) in approximately 30% of RAW264.7 cells at 24 hpi.

### *H. pylori* infection causes dysregulation of gene transcription in RAW264.7 cells

**Figure 2:**
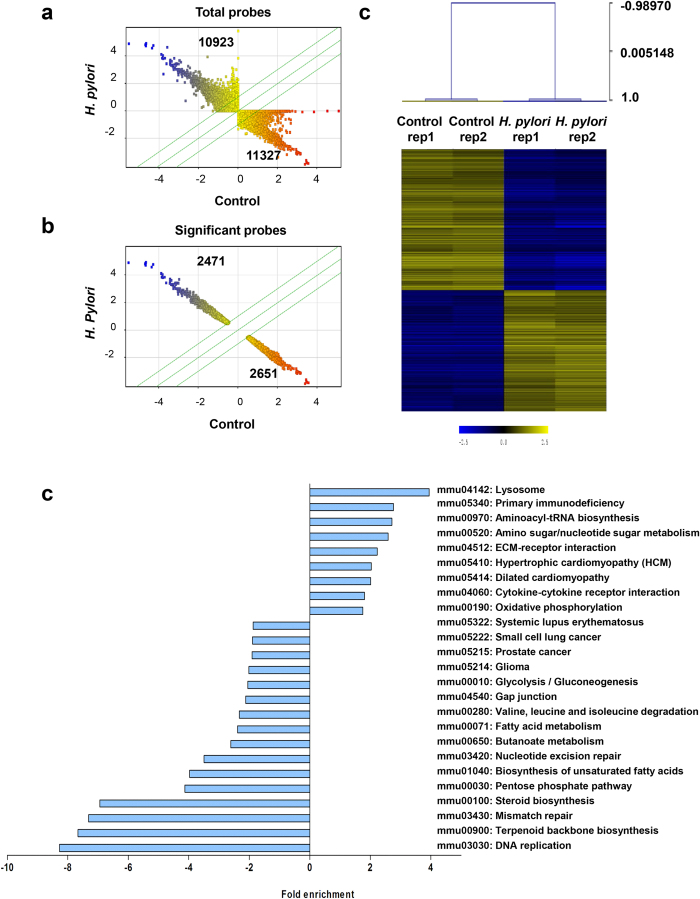
Microarray analysis of *H. pylori*-infected RAW264.7 cells. (**a** and **b**) Scatter plots show the expressions of total probes (**a**) and significant probes (**b**), in the non-infected control versus *H. pylori* (MOI 10, 24 h)-infected cells. Significant probes were selected based on FC < –2 or FC > 2, P < 0.05. X and Y axis show normalized log_2_ values. Number represents the number of probes in each quadrant. Blue and red dots show the intensities of up- or down-regulated probes, respectively based on the normalized values on X-axis. Three green lines demarcate the probes with FC of –2, 0 and 2. (**c**) Hierarchical clustering (HCL) for significant 5122 probes (2932 genes) was executed with Pearson Correlation distance metric and average linkage. (**d**) KEGG pathway analysis. Bar chart showing the FE of significantly modulated pathways in the *H. pylori*-infected RAW264.7 cells relative to control (FE < –2 or FE > 2, P < 0.05). In total, 8 pathways showed induction while 16 pathways were reduced.

We then compared the transcriptional milieu between uninfected and infected monocytic macrophages through a genome-wide microarray analysis. Two replicates of uninfected and *H. pylori* (MOI 10)-infected RAW264.7 cells for 24 h were prepared independently and analyzed on an Agilent SurePrint G3 Human GE 8 × 60k microarray platform which comprised 55,821 probes. Scatter plot was generated based on normalized (Log_2_) expression levels of total probes (Fig. 2a), and the total data were further filtered with fold changes (FC) > 2 or FC < –2 (^*^P > 0.05) to select significant differentially expressed probes (Fig. 2b). A total number of 2471 probes (1341 genes) and 2651 probes (1591 genes) were significantly up- and down-regulated, respectively. Using these significant probes, hierarchical clustering (HCL) was executed with Pearson Correlation distance metric and average linkage. Heat map generated showed two separate clusters (Fig. 2c), indicating that *H. pylori* infection influences the regulation of an array of genes in RAW264.7 cells in both upward and downward trends.

### DNA replication pathway is vitally subdued in *H. pylori*-infected RAW264.7 cells

Next, we performed pathway analysis using the Kyoto Encyclopedia of Genes and Genomes (KEGG) database on the genes whose expression was significantly altered by *H. pylori* infection and the fold enrichment (FE) score for each pathway was calculated. In total, 25 biological pathways with changes more than 2-fold (P < 0.05) were identified (Fig. 2d). Nine of these pathways were significantly enriched, among which lysosome pathway (mmu04142) showed the highest score (FE = 3.94, P < 0.0001) (Supplementary Table S1), followed by cytokine-cytokine receptor interaction pathway (mmu04060) (FE = 1.8, P = 0.0016). On the contrary, 16 pathways were significantly suppressed (Fig. 2d). Interestingly, DNA replication pathway (mmu03030) was the most suppressed pathway at FE = –8.27 (P < 0.0001) (Supplementary Table S1). Both mismatch (mmu03430) and nuclear excision (mmu03420) DNA repair mechanisms were identified at FE = –7.30 (P < 0.0001) and FE = –3.49 (P < 0.0001), respectively, consistent with previous studies reporting increased DNA damage and compromised DNA repair mechanism during *H. pylori* infection^22^,^23^. Notably, *H. pylori* infection significantly disrupted terpenoid backbone (mmu00900) and steroid (mmu00100) biosynthesis pathways (FE = –7.66, P < 0.0001, and FE = –6.93, P < 0.0001, respectively). Terpenoid precursors in eukaryotes through sterol biosynthetic pathways are converted to cholesterol, ergosterol and phytosterol that are crucial for antimicrobial activity^24^,^25^ and for cellular attachment by *H. pylori* and other intracellular pathogens^26^.

### Top 10 up- and down-regulated genes in *H. pylori*-infected RAW264.7 cells

The top 10 among the up- and down-regulated genes were identified (Table 1). The expression of the top 10 up- and down-regulated genes were verified by quantitative real-time-polymerase chain reaction (qRT-PCR) analysis (Supplementary Figure S2). Of the top 10 up-regulated genes, five of them were immune response-related genes that encode for cytokines *Csf1, Csf3*, and *Il-1β* and chemokines *Ccl7* and *Cxcl2. Csf1* and *Csf3* were significantly induced at 208-fold (P = 0.0002) and 771-fold (P = 0.0031), whereas the pro-inflammatory cytokine *Il-1β* was increased at 257-fold (P = 0.0028), relative to the uninfected control. *Csf1* (also known as *macrophage-colony stimulating factor*) and *Csf3* (also known as *granulocyte-colony stimulating factor*) are important factor for hematopoietic cells differentiation into macrophages and granulocytes, respectively^27^. *H. pylori* infection also elevated the transcription of chemokine genes *Ccl7* and *Cxcl2* by 682-fold (P = 0.0003) and 496-fold (P = 0.0008), respectively. *Ccl7* [previously known as *Monocyte-specific chemokine 3* (*Mcp3*)] is a chemoattractant for monocyte and macrophage^28^, whereas *Cxcl2* [previously named as *Macrophage inflammatory protein 2α* (*MIP2α*)] is chemotactic for polymorphonuclear leukocytes^29^. Thus, excessive expression of these cytokines and chemokines can intensify the immune responses at the infection site.

Three in the list of top 10 up-regulated genes were related to cell stress management, namely *Car6* (*Carbonic anhydrase 6*), *Pappa2* (*Pappalysin 2*) and *Ptgs2* (*Prostaglandin endoperoxidase synthase 2,* also named as *Cox2*)^30^,^31^,^32^. *Car6* was induced at 1396-fold (P = 0.0036), *Pappa2* at 233-fold (P = 0.0028) and *Ptgs2* at 541-fold (P = 0.0041). Interestingly, *Ptgs2* 5939C mutation has been shown to accelerate gastric carcinogenesis in the presence of an ongoing *H. pylori* infection^33^. Other genes in the top 10 up-regulated gene list were *Lcn2* (*Lipocalin 2*), induced by 746-fold (P = 0.0014) and *Avil* (*Advillin*) by 196-fold (P = 0.0003). *Lcn2* is a negative regulator of inflammation^34^ whereas *Avil* encodes for a member of gelsolin superfamily of actin binding protein^35^.

Meanwhile, top 10 down-regulated genes in *H. pylori*-infected RAW264.7 cells comprised of genes of diverse functions (Table 1). Expression of *Cx3cr1* was suppressed the greatest (C*hemokine C-X3-C receptor*, –157-fold, P = 0.0022). *Cx3cr1* plays a crucial role in luminal antigen sampling and clearance of entero-invasive pathogens. Hence, reduced *Cx3cr1* expression mediated by *H. pylori* could impair pathogen recognition by macrophages^36^. Intriguingly, two in the list were genes associated with cell cycle progress, i.e. *Aurkb* (*Aurora-B kinase*) and *Mybl2* (*Myeloblastosis oncogene-like 2,* also known as *b-myc*). Transcription of *Aurkb* and *Mybl2* genes were suppressed by 75- (P = 0.0017) and 66-fold (P = 0.0008), respectively. *Aurkb* is essential in chromatin protein modification and G1-to-S phase transition^37^,^38^ while *Mybl2* regulates transcription of various cell cycle genes such as *Cyclin D1*, *Cyclin B2*, *C-myc* and *Cdc25b*^39^.

*Ankle1* (*Ankyrin repeat and LEM domain containing 1*), an essential molecule in DNA cleavage and DNA damage response^40^, was reduced by 71-fold (P = 0.0002). Other genes in the list were *Fgd2* (*FYVE, RhoGEF and PH domain containing 2*, –146-fold, P = 0.0021), *Kbtbd11* (*Kelch repeat and BTB (POZ) domain containing 11*, –133-fold, P = 0.0007), *Dhcr24* (*24-dehydrocholesterol reductase*, –78-fold, P = 0.0001), *Alox5* (*Arachidonate 5-lipoxygenase*, –78-fold, P = 0.0002), *Lrp8* (*Low density lipoprotein receptor-related protein 8*, –70-fold, P = 0.0008), and *Crip1* (*Cysteine-rich protein 1*, –67-fold, P = 0.0006). *Fgd2* is a CDC42-specific exchange factor that activates membrane ruffles in antigen presenting cells. *Dhcr24* is crucial for cholesterol synthesis and has a pro-survival role during oxidative stress^41^,^42^*. Alox5* and *Lrp8* polymorphisms have been linked to coronary artery disease and osteoblast differentiation^43^,^44^,^45^,^46^ whereas the functions for *Kbtbd11* and *Crip1* are not well-defined.

### *H. pylori* infection activates immune response-related genes in RAW264.7 cells

**Figure 3:**
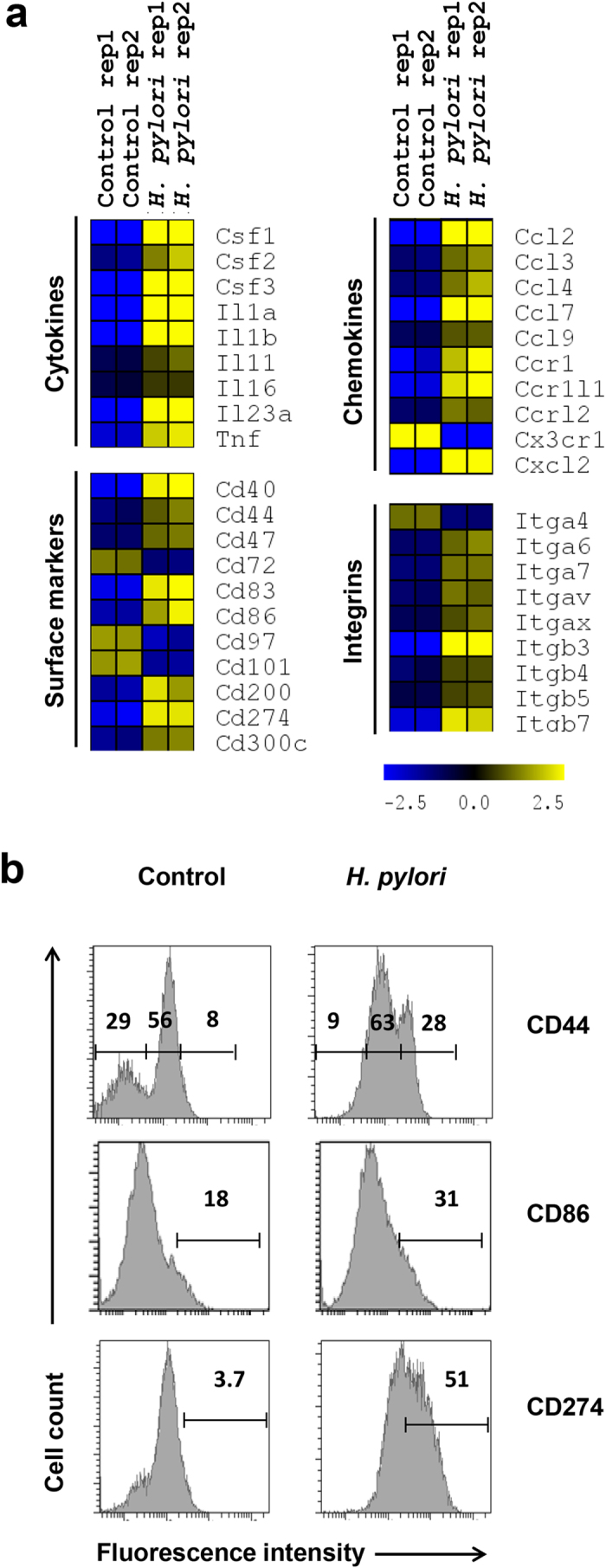
*H. pylori* infection up-regulates genes encoded for immune reactions. (**a**) Heatmap of significant genes encoded for cytokines, surface markers, chemokines, and intergrins. Color intensity reflects the normalized log_2_ values of the RNA abundance. Yellow: increase, blue: decrease, dark: no change. (**b**) Flow cytometrical analysis of cell surface markers on the control and *H. pylori* (MOI 10, 24 h)-infected cells. Fluorescence intensities for different markers were as shown. Numbers represent the percentages of cell in the gated area.

To identify the biological functions of genes with pronounced expression changes subsequent to *H. pylori* infection, we categorized the differentially expressed genes according to their functional groups. As anticipated, majority of the immune-associated genes were markedly up-regulated by *H. pylori* infection (Fig. 3a). Colony stimulating factors *Csf1*, *Csf2* and *Csf3*, were considerably up-regulated, along with pro-inflammatory cytokines *Il1α*, *Il1β,* and *Tnfα*, suggesting that *H. pylori*-infected RAW264.7 cells may trigger macrophage and granulocyte differentiation and promote robust inflammatory responses^27^. Further, up-regulation of *Il23α* in *H. pylori*-infected RAW264.7 cells may hasten the differentiation of T helper 17 cells to combat against *H. pylori*^47^. Activation markers including *Cd44*, *Cd40*, *Cd86*, and *Cd274* were greatly up-regulated in *H. pylori*-infected macrophages. CD44 is a cell surface glycoprotein important for interaction and adhesion while CD40 and CD86 are receptor ligands for T cell CD40L, and CD28/CTLA4, respectively. Cd274 (also known as programmed cell death-1 ligand, PD-1L) binds to PD-1 receptor to modulate cell activation and inhibition^48^. Using flow cytometrical analyses, we verified that these transcriptional regulations were translated at protein levels (Fig. 3b). Shift of *H. pylori*-infected RAW264.7 cells from CD44^low/medium^ into CD44^medium/high^ population suggests cellular activation. Expression of CD86 was 13% higher in *H. pylori*-infected cells than in uninfected population, and 51% of the infected cells expressed CD274 compared to only 3.7% of the uninfected cells.

Conversely, the expression of *Cd72*, *Cd97* and *Cd101* were substantially down-regulated. CD72 and CD101 are known negative regulators of lymphocyte function. The cytoplasmic domain of CD72 consists of an immunoreceptor tyrosine inhibitor motif (ITIM) that suppresses B cell maturation and plasma cell differentiation^49^,^50^ whereas CD101 (V7) inhibits T cell proliferation and T cell receptor signaling^51^,^52^. The function of CD97, an adhesion-linked G-protein-coupled receptor in immune cells remains poorly defined^53^. Besides, multiple genes encoding for chemokines/chemokine receptors (*Ccl2*, *Ccl7*, *Ccr1*, *Cxcl12,* etc.) and integrins (*Itgβ3*, *Itgβ7*, etc.) were greatly up-regulated. Together, these data suggest that *H. pylori* triggers robust transcription of genes that culminated in the activation of RAW264.7 cells.

### *H. pylori* infection suppresses transcription of genes encoding for DNA synthesis and cell cycle progress

**Figure 4:**
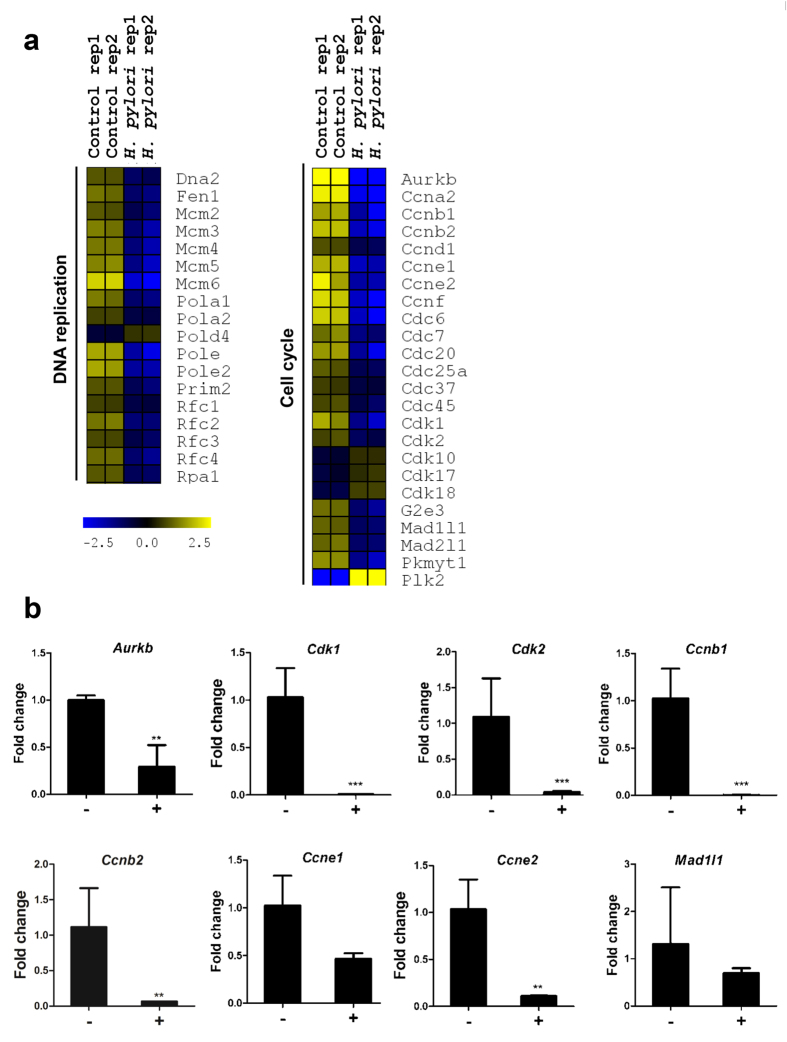
*H. pylori* infection down-regulates genes encoded for DNA synthesis and cell cycle molecules. (**a**) Heatmap of significant genes encoded for DNA synthesis and cell cycle molecules. Color intensity reflects the normalized log_2_ values of the RNA abundance. Yellow: increase, blue: decrease, dark: no change. (**b**) qRT-PCR analysis. Relative fold change shows expression of each gene relative to internal control β-actin. –: Non-infected control; +: *H. pylori* (MOI 10, 24 h)-infected cells. Data were shown as mean ± SD, from one experiment run in triplicate. Statistical significance was analyzed with unpaired Student’s *t*-test (*P < 0.05, **P < 0.01, ***P < 0.001).

In contrast to the enhanced expression of immune response-related genes, majority of genes involved in DNA replication such as members of *Mcm* (*minichromosome maintenance*), *Pol* (*DNA polymerase*), and *Rfc* (*replication factor C*) families were substantially down-regulated (Fig. 4a). Genes encoding for cyclins (*Ccna1*, *Ccnb1*, etc.), cyclin-dependent kinases (*Cdk1* and *Cdk2*) and mitotic arrest deficient-like proteins (*Mad1l1* and *Mad1l2*) were similarly down-regulated. The expression of selected genes that were altered consequent to *H. pylori* infection were verified by qRT-PCR (Fig. 4b). Consistent with the microarray data, *Aurkb* expression was reduced by 3.4 times following *H. pylori* infection. The mRNA levels of *Ccnb1*, *Ccnb2, Ccne1,* and *Ccne2* were reduced by 201-, 17-fold, 2.2- and 9.4-fold, respectively. Likewise, *Cdk1* and *Cdk2* expression decreased by 112- and 27-fold, respectively, while *Mad1l1* was reduced by 1.8-fold.

### *H. pylori* blocks G1-to-S transition by suppressing Aurkb activity

**Figure 5:**
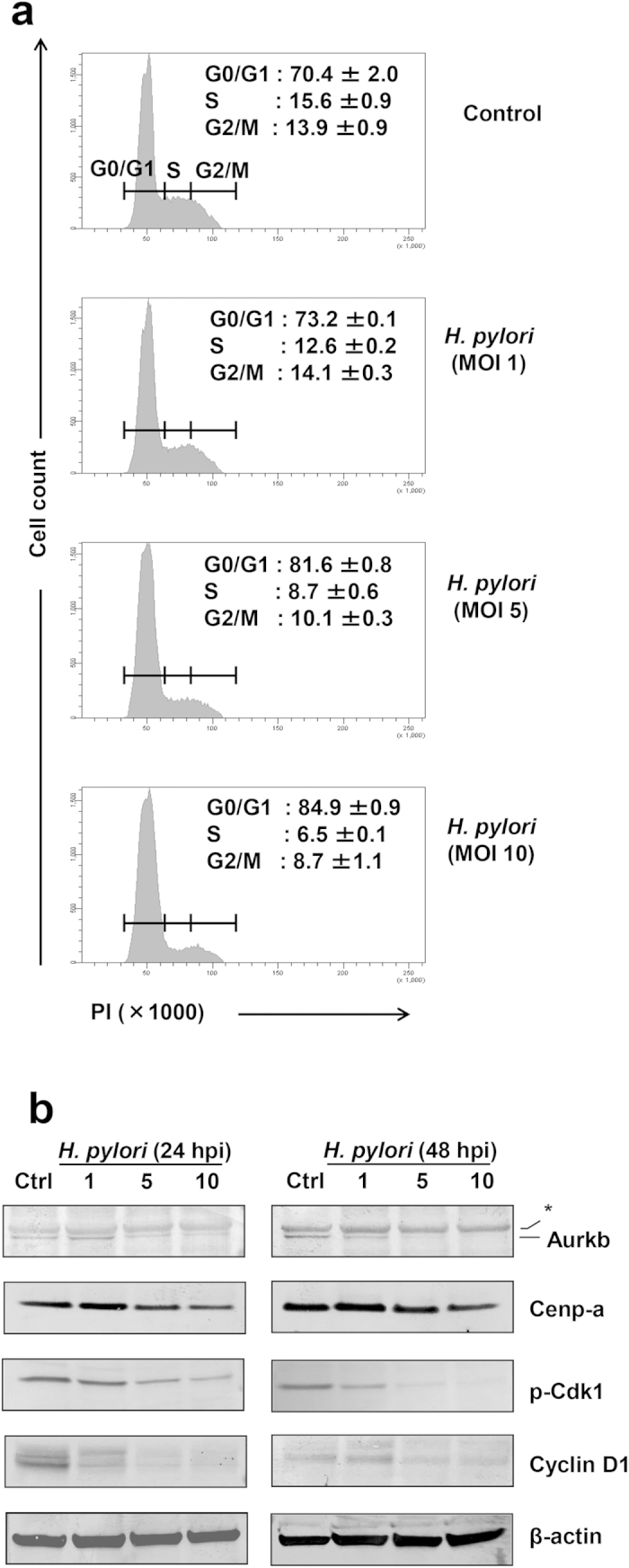
*H. pylori* infection blocks cell cycle progress. (**a**) Cell cycle analysis of control and *H. pylori* (MOI 1, 5 or 10)-infected RAW264.7 cells. Cells were harvested at 24 hpi, fixed and stained with propidium iodide (PI) to detect DNA contents. Numbers represent the percentages of cells at G0/G1, S or G2/M phases. Data were shown as mean ± SD from one experiment run in duplicate, and were representative data of two independent experiments. (**b**) Immunoblot analysis of cell lysates prepared from control and *H. pylori*-infected RAW264.7 cells for 24 or 48 h. Antibodies against AurkB, Cenp-a, phospho-Cdk1 (Cdc2) or Cyclin D1 were used. β-actin was used as loading control. *All gels were run under same experimental condition. Images were cropped from full length blots (Supplementary Figure S4). Shown are representative data of two independent experiments.

Given that *H. pylori* infection of macrophages impaired multiple genes associated with DNA replication and cell cycle, we performed propidium iodide staining and flow cytometrical analysis of the infected RAW264.7 cells to examine whether dysregulated cell cycle gene transcriptions would impact cell cycle progress (Fig. 5a). Percentage of cells at the S phase were reduced by approximately 3-fold upon *H. pylori* infection (6.5 ± 0.1% versus 15.6 ± 0.9% in controls). Additionally, the proportion of infected cells at G2/M phase were reduced by almost 50% (8.7 ± 1.1% versus 13.9 ± 0.9% in controls). These observations were accompanied by an increased number of cells retained at the G0/G1 phase (from 70.4 ± 2.0% to 84.9 ± 0.9%).

During cell cycle, AurkB mediates S phase entry by interacting with Cdk1 (Cdc2)^54^. Through immunoblot analysis, we found that AurkB protein level was diminished in cells infected with *H. pylori* (Fig. 5b). Concurrently, AurkB-mediated downstream phosphorylation of Cdk1 was also abolished (Fig. 5b). In addition to mediating S phase entry, AurkB initiates G2/M transition by activating kinetochore protein complexes including Cenp-a^37^. We observed that the mitotic protein Cenp-a was reduced in *H. pylori*-infected cells. The reduced levels of *Cenp-*a and other mitosis-related proteins (*Espl1* and *Zwilch*) were also detected at transcriptional level (Supplemental Figure S2). In addition, expression of Cyclin D1 was suppressed in *H. pylori*-infected RAW264.7 cells. These data suggest that *H. pylori* could block G1/S and G2/M transitions by inhibiting formation of AurkB and cyclin/cdk complexes.

### *H. pylori* infection attenuates proliferation of RAW264.7

**Figure 6:**
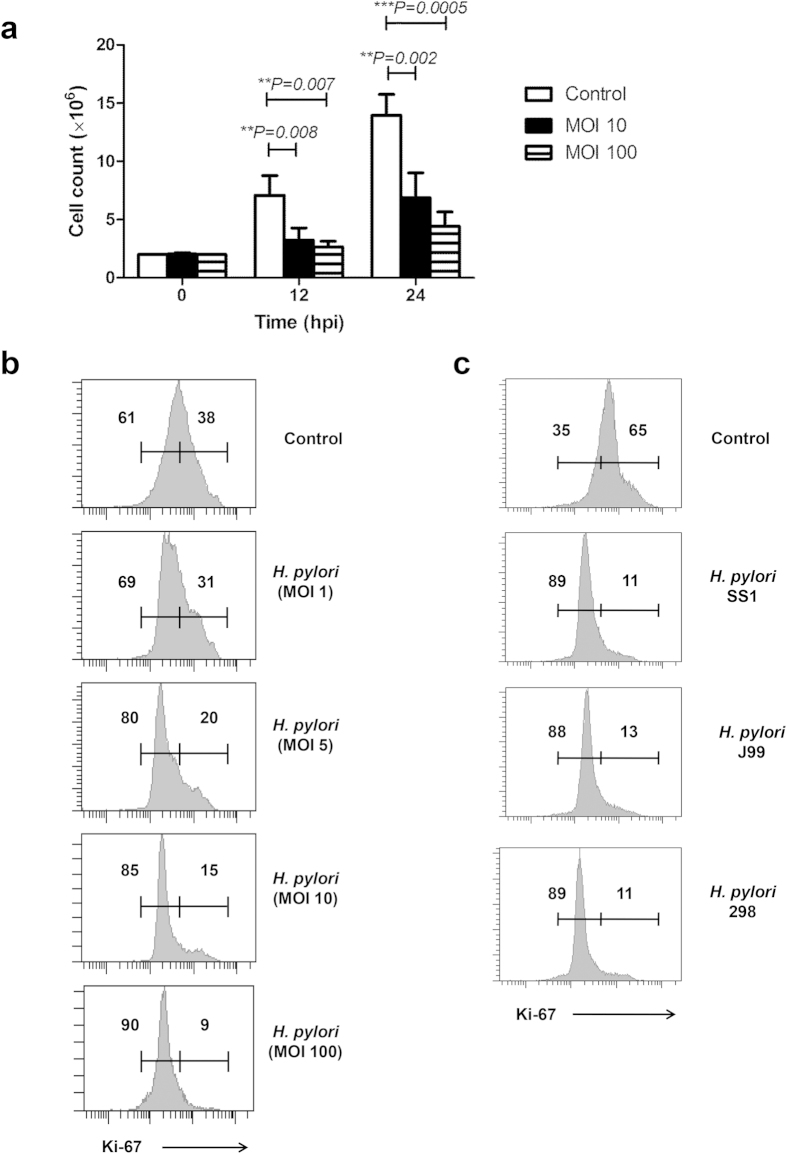
Effect of *H. pylori* infection on the cell proliferation of RAW264.7 cells. (**a**) Bar chart shows absolute cell count of the non-infected and *H. pylori*-infected cells at 12 or 24 hpi. Data were shown as mean ± SD, from one experiment run in triplicate. Statistical significance were analyzed with unpaired Student’s *t*-test (**P < 0.01, ***P < 0.001). (**b** and **c**) Flow cytometrical analysis of intranuclear expression of Ki-67 cell proliferation marker in the control and *H. pylori*-infected cells. (**b**) RAW264.7 cells were infected with 1, 5, 10 and 100 MOIs of *H. pylori* SS1 strain for 24 h. (**c**) RAW264.7 cells were infected with different strains of *H. pylori* including SS1, J99 and 298 at MOI 100 for 24 h. Numbers represent the percentages of cells in the gated area. Shown were representative data of two independent experiments.

Next, we examined the proliferative activity of *H. pylori*-infected RAW264.7 cells (Fig. 6). Consistent with cell cycle blockage, we observed reduced mitotic division in *H. pylori*-infected cell population. Absolute cell count likewise revealed 50% reduction in cell number among infected macrophages. At 24 hpi, macrophage count of the infected 6.8 ± 1.8 million cells to 3.2 ± 0.9 million cells (P = 0.007) while at 48 hpi, the count decreased from 13.9 ± 1.6 million cells to 7.1 ± 1.5 million cells (P = 0.002) (Fig. 6a). Accordingly, the fraction of infected cells expressing intranuclear proliferation marker Ki-67 shrunk by 15%, accompanied by an increase of non-proliferative Ki-67^low^ RAW264.7 population (Fig. 6b). Collectively, these data suggest that *H. pylori* infection inhibits proliferation of RAW264.7 cells.

### Both CagA+ or CagA-deficient *H. pylori* strains cause anti-proliferative effect in RAW264.7 cells

*H. pylori* produces cytotoxin CagA and VacA which can destroy the gastric epithelium and lead to ulcer formation^55^,^56^. *H. pylori* SS1 is a mouse-adapted strain^57^ which is deficient in the function of Cag pathogenicity island (PAI)^58^ and possesses VacA s2m2 genotype. Next, we examined two other *H. pylori* strains with functional CagPAI and different vacA genotype for ability to induce anti-proliferative effect in the RAW264.7 cells. To address this, we infected cells with two additional *H. pylori* strains, namely J99 and 298. Both *H. pylori* J99 (a standard strain) and 298 (a mice-adapted derivative from a local clinical isolate - UM032) have complete CagPAI, CagA and the more cytotoxic VacA s1m1 genotype^59^,^60^. Our results showed that infection of RAW264.7 cells with SS1, J99 or 298 demonstrated comparable cell proliferation (Fig. 6c) and cell cycle effects (Supplementary Figure S3), suggesting that *H. pylori* effectively inhibited the RAW264.7 cell proliferation, regardless of CagPAI or VacA activities.

### *H. pylori* infection attenuates proliferation of primary macrophage cells

**Figure 7:**
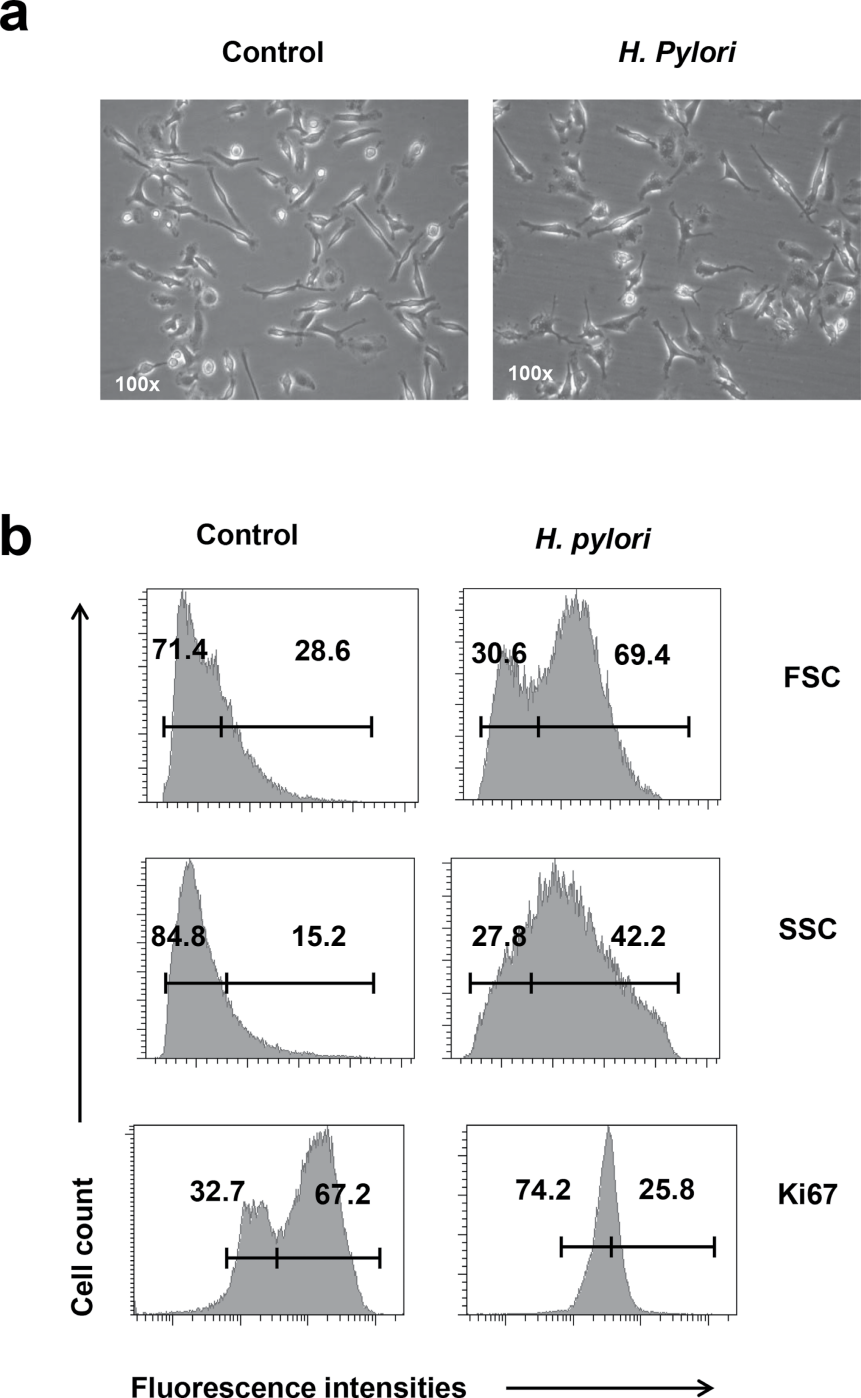
Effect of *H. pylori* on the cell proliferation of primary macrophage cells. BMDM cells were prepared by stimulating the C57BL/6 mice bone marrow cells with M-CSF (20 ng/ml) for 7 days. BMDM cells were infected with MOI 10 of *H. pylori* SS1 strain for 24 h. (**a**) Photos of the non-infected and *H. pylori* SS1 (MOI 10)-infected BMDM cells. (**b**) Flow cytometrical analysis of forward scatter (FSC), side scatter (SSC) and intranuclear expression of Ki-67 cell proliferation marker in the control and *H. pylori*-infected cells. Numbers represent the percentages of cells in the gated area. Shown were representative data of two independent experiments.

Because the above assays were performed using RAW264.7 cell line, we would like to use primary macrophage cells, to confirm the infectivity and anti-proliferative effect of *H. pylori* in the macrophages. Bone marrow cells were isolated from C57BL/6 mice and stimulated with 20 ng/ml M-CSF for 7 days to obtain bone marrow-derived macrophage (BMDM) cells. *H. pylori* infection for 24 h resulted in decreased proliferative cells within BMDM population (Fig. 7), supporting the ability of *H. pylori* to effectively block the macrophage cell proliferation.

## Discussion

*H. pylori* is a Gram-negative bacterium colonizing nearly half of the human population and is a well-established etiological agent of gastritis, peptic ulcer and gastric cancer. In this study, we reported disrupted gene transcriptional program in *H. pylori*-infected RAW264.7 monocytic macrophage cells. Consequently, cell cycle progression and proliferation of these infected macrophages were greatly suppressed. *H. pylori* infection shifted cells into Ki-67^low^ non-proliferative stage, arrested cells at G0/G1 phases, and impeded entry of cells into S or G2/M phases. To date, there is no evidence showing that *H. pylori* causes cell cycle arrest in immune cells, although *H. pylori* has been reported to retain epithelial cells at G1/S and G2/M phases through increasing p27Kip1 and decreasing cyclin E/Cdk2 complex activities^61^. Besides, the *H. pylori* L-asparaginase can function as a cell cycle inhibitor by preventing entry into S phase in gastric cells^62^.

The cell cycle progression through G1/S checkpoint is controlled by sequential activation of cyclin/Cdk complexes^63^. At G1 phase, D-type cyclins (D1, D2 and D3) associate with Cdk4/Cdk6, while Cyclin E forms a complex with Cdk2. These cyclin-Cdk complexes sequentially activate retinoblastoma (Rb) protein and E2F that control the expression of a cluster of S phase-associated genes including Cyclin A and Cdk1^64^. During the G1-to-S transition, AurkB kinase plays an essential role in augmenting phosphorylation and activation of Rb, Cdk1 and Cdk2^38^. Our study showed that *H. pylori*-mediated transcriptional inhibition of genes encoding for *Cyclin D1*, *Cdk1* and *Cdk2* coupled with suppression of AurkB and Cdk1 phosphorylation may halt G1-to-S transition. Doubling of DNA content via DNA replication occurs during the S phase prior to M phase. During this stage, members of Mcm family form pre-replication complex that unravels the double helix and initiates replication at early S phase^65^,^66^ whereas the Pol^67^,^68^ and Rfc^69^ complexes are important for DNA elongation. Strikingly, we observed that expression of numerous genes within the Mcm, Pol and Rfc families were strongly down-regulated in the *H. pylori*-infected RAW264.7 cells. Furthermore, KEGG pathway analysis showing depletion of DNA replication pathway upon *H. pylori* infection supports the hypothesis that *H. pylori* blocks DNA replication and inhibits cell cycle at S phase.

It is important to note that the *H. pylori*-mediated cell cycle inhibition is not limited to G1-to-S transition, but it occurs simultaneously at G2/M phase. This is supported by cell cycle assay and microarray data which showed significant suppression by *H. pylori* infection of multiple genes encoding for molecules associated with G2/M progress. AurkB may also play a key role in this process because in addition to regulating G1-to-S transition^38^, it participates in mitosis by modulating spindle function^70^ in which AurkB controls centromere protein complex that includes histone Cenp-a protein which is responsible for assembly of kinetochore proteins^71^,^72^. Moreover, expression of Mad1l1, a protein vital for mitosis progression and checkpoint control, is reduced by *H. pylori*^73^,^74^. In our study, *H. pylori*-infected RAW264.7 cells exhibited reduced mRNA and protein levels of AurkB and its substrate Cenp-a. In addition, genes encoding for Zwilch kinetochore proteins and for Separase (encoded by *Espl1*) indispensable for anaphase spindle elongation^75^ were significantly suppressed by *H. pylori* (Supplementary Figure S2). Therefore, disruption of these processes can result in mitotic checkpoint failure, resulting in premature mitotic exit and chromosome mis-segregation that underlie tumorigenesis.

Macrophage is a key player in *H. pylori* pathogenesis in which its depletion causes reduced pathology in the gastric^3^. One of the major roles of macrophage is to trigger adaptive immune response. *H. pylori*-infected macrophage is able to produce BAFF which promotes T_H_17 cell expansion by creating a pro-T_H_17 milieu or by direct control of naïve T cell differentiation^76^. The ability of *H. pylori* to form chronic colonization in the host relies on their effective immune evasion strategies^77^. Previous study showed the *H. pylori* infection in macrophage can cause cell death by induction of macrophage arginase II^17^. In this study, we suggest a different strategy by which the *H. pylori* is able to block various cell proliferation-associated genes thus inhibits the macrophage cell growth. In addition to inhibiting macrophage, *H. pylori* VacA exotoxin interferes with the T cell activation through inhibiting calcium influx thus preventing NFAT nuclear translocation and the subsequent cytokine transactivation^78^. Besides, VacA is also able to interfere with antigen presentation by major histocompatibility complex in B cells^79^.

In summary, we observed that *H. pylori* infection impaired mitotic proliferation of RAW264.7 monocytic macrophage cells. G1-to-S cell cycle transition was inhibited in the infected cells subsequent to depleted expression of AurkB- and cyclins/cdks-encoding genes. We anticipate that *H. pylori*-mediated interference of macrophage proliferation is possibly one of the strategies employed by *H. pylori* to limit the quantity of macrophages at the infection site and to evade efficient clearance by host immune system.

## Methods

### Bacteria

A mouse-adapted strain of *H. pylori* SS1 strain^57^, was provided by the *H. pylori* Research Laboratory, University of Western Australia. J99 strain was from Amerian Type Culture Collection (ATCC, Rockwille, MA)^59^ while 298 strain was derived from a local clinical isolate, UM032, as previously described^60^. Bacteria was grown on chocolate agar plate supplemented with 7% laked horse blood (Oxoid, Basingstoke, UK) under microaerophilic conditions at 10% CO_2_, 37 °C in a humidified incubator and were subcultured every 3 days. For infection, *H. pylori* was harvested in brain heart infusion (BHI) broth and quantified by a spectrophotometer (OD_650 nm_ of 1 = 1 × 10^8^ cells/ml). Viable cell count was predetermined by calculating colony forming units after serially diluted bacteria were drop plated onto chocolate agar plate.

### Tissue culture

RAW264.7 cells were purchased from America Type Culture Control (ATCC TIB-71). RAW264.7 cells were cultured in Dulbecco’s Modified Essential Medium supplemented with 10% heat inactivated fetal bovine serum and incubated at 37^o^ C, 5% CO_2_. One day prior to inoculation, cells were seeded in a T25 flask at 5 × 10^5^ cells/ml. Cells were then infected with *H. pylori* SS1 at MOIs of 1, 5 or 10 for 24 h.

### Primary macrophage cell preparation

C57BL/6 mice were purchased (Jackson Laboratory, Bar Harbor, ME). Preparation of primary macrophages was adapted from a previous report^80^. Two male mice at 8–12 weeks old were euthanized and bone marrow cells were isolated from the femurs. Cells were cultured in RPMI 1640 medium supplemented with 10% heat-inactivated FBS, 100 μg/ml streptomycin and 100 U/ml penicillin, 1× non-essential amino acids, 1 mM HEPES and stimulated with 20 ng/ml M-CSF (Biolegend, San Diego, CA). After 3 days, non-adherent cells were collected and cultured for another 3 days to obtain BMDM^81^. At day 7, adherent cells were infected with *H. pylori* SS1 at MOI 10 for 24 h.

### RNA extraction and qRT-PCR

RNA was isolated from cells using TRIzol reagent (Invitrogen, Carlsbard, CA) as described^82^. RNA integrity number (RIN) was >9.5 as determined using Bioanalyzer 2100. cDNA was prepared using M-MLV reverse transcriptase (Invitrogen). qRT-PCR was carried out with SsoAdvanced SYBR Green Supermix (Biorad, Hercules, CA) in a Real-Time PCR 7500 (Applied Biosystems, Foster City, CA) using designed primers (Supplementary Table S2). Relative fold change was calculated using comparative 2^−ΔΔCT^ method. All experiments were run in triplicates and were presented as mean ±SD.

### Microarray analysis

Microarray analysis was performed with Agilent Technologies microarray platform using Agilent SurePrint G3 Human GE 8 60k containing 55,821 probes (Design ID: G4851A, Lot: 0006097429). Total RNA (100 ng) was primed with an oligo-dT containing the recognition site for RNA polymerase. RNA was labelled using Low Input Quick Amp Labeling Kit, One-Color (Agilent p/n 5190-2305) to produce cyanine 3-CTP labeled cRNA. cRNA (600 ng) was hybridized onto 8-array slide at10 rpm for 17 h at 65 °C. The slide was washed and scanned on Agilent High Resolution Microarray Scanner (C-model). Raw signal data were extracted from the TIFF image with Agilent Feature Extraction Software (V107.1.1). Pathway analysis was performed using the KEGG database^83^,^84^. Heat maps were generated with multiexperimental viewer (MeV) software^85^.

### Flow cytometry analyses

Cells (1 × 10^6^) were stained with antibodies for 30 min in dark before analyzed in a FACS Canto cytometer (BD Biosciences, Franklin Lakes, NJ). For intranuclear staining, cells were fixed and permeabilized using Foxp3/transcription factor staining buffer set (eBioscience, San Diego, CA) and stained with Ki67. The antibodies used included PE-conjugated PD-1L, PerCPCy5.5-conjugated F4/80, APC-conjugated CD11b (Mac-1), CD44 and CD86 (Biolegend). For cell cycle, cells were fixed overnight in 70% ice-cold ethanol at –80 °C, as described^86^. Cells were stained with propidium iodide/ribonuclease A (RNaseA) solution (BD Biosciences) for 30 min before analyzed using flow cytometer.

### Immunoblot analysis

Cell lysates were prepared in RIPA lysis buffer (Santa Cruz Biotech, Santa Cruz, CA), separated by NuPage gel (Invitrogen) and blotted onto polyvinylidenedifluoride membranes. Membranes were blocked with 5% BSA in TBS-T and incubated with primary (1:1,000) and secondary (1:5,000) antibodies. Primary antibodies used were antibodies against β-actin, Cyclin D1 (DCS6), phospho-Cdc2 (Tyr15), Cenp-a (Cell Signaling Technologies, Beverly, MA) and Aurora-B kinase (Abcam, Cambridge, UK). Secondary antibodies used were alkaline phosphatase-conjugated mouse or rabbit anti-IgG (Promega, Madison, WI). Membranes were developed using colorimetric NBT-BCIP substrate (Promega), as described^87^.

### Statistical analysis

Data were analyzed with unpaired two-tailed Student’s *t*-test or Benjamini-Horchberg False Discovery Rate (FDR) multiple testing correction. Samples were considered significant if P < 0.05.

## Additional Information

**How to cite this article**: Tan, G. M. Y. *et al.* Suppression of cell division-associated genes *by Helicobacter pylori* attenuates proliferation of RAW264.7 monocytic macrophage cells. *Sci. Rep.* **5**, 11046; doi: 10.1038/srep11046 (2015).

## Supplementary Material

Supplementary Information

## Figures and Tables

**Table 1:** List of top 10 up- and down-regulated genes in *H. pylori-*infected RAW264.7 cells.

**Genbank Accession**	**Description**	**Folds**	**P**	**FDR**
**Up-regulation**				
NM_009802	*Carbonic anhydrase 6 (Car6)*	1396	0.0036	0.0291
NM_009971	*Colony stimulating factor 3(Csf3)*	771	0.0031	0.0279
NM_008491	*Lipocalin 2 (Lcn2)*	746	0.0014	0.0238
NM_013654	*Chemokine (C-C motif) ligand 7 (Ccl7)*	682	0.0003	0.0209
NM_011198	*Prostaglandin-endoperoxide synthase 2 (Ptgs2)*	541	0.0041	0.0984
NM_009140	*Chemokine (C-X-C motif) ligand 2 (Cxcl2)*	496	0.0008	0.0216
NM_008361	*Interleukin 1 beta (Il1b)*	257	0.0064	0.0345
NM_001085376	*Pappalysin 2 (Pappa2)*	233	0.0028	0.0273
NM_007778	*Colony stimulating factor 1 (Csf1)*	208	0.0002	0.0209
NM_009635	*Advillin (Avil)*	196	0.0003	0.0209
**Down-regulation**				
NM_009987	*Chemokine (C-X3-C) receptor 1 (Cx3cr1)*	−157	0.0022	0.0259
NM_013710	*FYVE, RhoGEF and PH domain containing 2 (Fgd2)*	−146	0.0021	0.0255
NM_029116	*Kelch repeat and BTB (POZ) domain containing 11 (Kbtbd11)*	−133	0.0007	0.0211
NM_053272	*24-dehydrocholesterol reductase (Dhcr24)*	−78	0.0001	0.0145
NM_009662	*Arachidonate 5-lipoxygenase (Alox5)*	−78	0.0002	0.0210
NM_011496	*Aurora kinase B (Aurkb)*	−75	0.0017	0.0246
NM_172756	*Ankyrin repeat and LEM domain containing 1 (Ankle1)*	−71	0.0002	0.0210
NM_001080926	*Low density lipoprotein receptor-related protein 8, apolipoprotein e receptor (Lrp8)*	−70	0.0008	0.0213
NM_007763	*Cysteine-rich protein 1 (intestinal) (Crip1)*	−67	0.0006	0.0145
NM_008652	*Myeloblastosis oncogene-like 2 (Mybl2)*	−66	0.0008	0.0216

Significance analysis was performed with Student’s *t*-test and Benjamini-Horchberg False Discovery Rate (FDR) multiple testing correction.

## References

[b1] Pathak S. K., Tavares R., de Klerk N., Spetz A. L. & Jonsson A. B. Helicobacter pylori protein JHP0290 binds to multiple cell types and induces macrophage apoptosis via tumor necrosis factor (TNF)-dependent and independent pathways. PloS one 8, e77872, 10.1371/journal.pone.0077872 (2013). 24223737 PMC3815203

[b2] Peek R. M. Jr., Fiske C. & Wilson K. T. Role of innate immunity in Helicobacter pylori-induced gastric malignancy. Physiol. Rev. 90, 831–858, 10.1152/physrev.00039.2009 (2010). 20664074 PMC2990353

[b3] Kaparakis M. *et al.* Macrophages are mediators of gastritis in acute Helicobacter pylori infection in C57BL/6 mice. Infect. Immun. 76, 2235–2239, 10.1128/IAI.01481-07 (2008). 18332213 PMC2346689

[b4] Krauss-Etschmann S. *et al.* Increase of antigen-presenting cells in the gastric mucosa of Helicobacter pylori-infected children. Helicobacter 10, 214–222, 10.1111/j.1523-5378.2005.00313.x (2005). 15904479

[b5] Craig P. M., Territo M. C., Karnes W. E. & Walsh J. H. Helicobacter pylori secretes a chemotactic factor for monocytes and neutrophils. Gut 33, 1020–1023 (1992). 1398224 10.1136/gut.33.8.1020 PMC1379434

[b6] Mai U. E. *et al.* Surface proteins from Helicobacter pylori exhibit chemotactic activity for human leukocytes and are present in gastric mucosa. J. Exp. Med. 175, 517–525 (1992). 1732414 10.1084/jem.175.2.517 PMC2119134

[b7] Ito T. *et al.* Helicobacter pylori invades the gastric mucosa and translocates to the gastric lymph nodes. Lab. Invest. 88, 664–681, 10.1038/labinvest.2008.33 (2008). 18475258

[b8] Whitney A. E. *et al.* Increased macrophage infiltration of gastric mucosa in Helicobacter pylori-infected children. Dig. Dis. Sci. 45, 1337–1342 (2000). 10961712 10.1023/a:1005551903029

[b9] Fehlings M. *et al.* Comparative analysis of the interaction of Helicobacter pylori with human dendritic cells, macrophages, and monocytes. Infect. Immun. 80, 2724–2734, 10.1128/IAI.00381-12 (2012). 22615251 PMC3434561

[b10] Quiding-Jarbrink M., Raghavan S. & Sundquist M. Enhanced M1 macrophage polarization in human helicobacter pylori-associated atrophic gastritis and in vaccinated mice. PloS one 5, e15018, 10.1371/journal.pone.0015018 (2010). 21124899 PMC2990716

[b11] Allen L. A., Schlesinger L. S. & Kang B. Virulent strains of Helicobacter pylori demonstrate delayed phagocytosis and stimulate homotypic phagosome fusion in macrophages. J. Exp. Med. 191, 115–128 (2000). 10620610 10.1084/jem.191.1.115 PMC2195807

[b12] Ramarao N., Gray-Owen S. D., Backert S. & Meyer T. F. Helicobacter pylori inhibits phagocytosis by professional phagocytes involving type IV secretion components. Mol. Microbiol. 37, 1389–1404 (2000). 10998171 10.1046/j.1365-2958.2000.02089.x

[b13] Mitchell P. *et al.* Chronic exposure to Helicobacter pylori impairs dendritic cell function and inhibits Th1 development. Infect. Immun. 75, 810–819, 10.1128/IAI.00228-06 (2007). 17101659 PMC1828488

[b14] Galgani M. *et al.* Helicobacter pylori induces apoptosis of human monocytes but not monocyte-derived dendritic cells: role of the cag pathogenicity island. Infect. Immun. 72, 4480–4485, 10.1128/IAI.72.8.4480-4485.2004 (2004). 15271906 PMC470697

[b15] Asim M. *et al.* Helicobacter pylori induces ERK-dependent formation of a phospho-c-Fos c-Jun activator protein-1 complex that causes apoptosis in macrophages. J. Biol. Chem. 285, 20343–20357, 10.1074/jbc.M110.116988 (2010). 20410304 PMC2888446

[b16] Gobert A. P. *et al.* Helicobacter pylori induces macrophage apoptosis by activation of arginase II. J. Immunol. 168, 4692–4700 (2002). 11971019 10.4049/jimmunol.168.9.4692

[b17] Lewis N. D. *et al.* Immune evasion by Helicobacter pylori is mediated by induction of macrophage arginase II. J. Immunol. 186, 3632–3641, 10.4049/jimmunol.1003431 (2011). 21296975 PMC3069806

[b18] Chaturvedi R. *et al.* Induction of polyamine oxidase 1 by Helicobacter pylori causes macrophage apoptosis by hydrogen peroxide release and mitochondrial membrane depolarization. J. Biol. Chem. 279, 40161–40173, 10.1074/jbc.M401370200 (2004). 15247269

[b19] Menaker R. J., Ceponis P. J. & Jones N. L. Helicobacter pylori induces apoptosis of macrophages in association with alterations in the mitochondrial pathway. Infect. Immun. 72, 2889–2898 (2004). 15102801 10.1128/IAI.72.5.2889-2898.2004 PMC387848

[b20] Wang Y. H., Wu J. J. & Lei H. Y. The autophagic induction in Helicobacter pylori-infected macrophage. Exp. Biol. Med. 234, 171–180, 10.3181/0808-RM-252 (2009). 19064937

[b21] Bussiere F. I. *et al.* Low multiplicity of infection of Helicobacter pylori suppresses apoptosis of B lymphocytes. Cancer Res. 66, 6834–6842, 10.1158/0008-5472.CAN-05-4197 (2006). 16818661

[b22] Kim J. J. *et al.* Helicobacter pylori impairs DNA mismatch repair in gastric epithelial cells. Gastroenterology 123, 542–553 (2002). 12145807 10.1053/gast.2002.34751

[b23] Obst B., Wagner S., Sewing K. F. & Beil W. Helicobacter pylori causes DNA damage in gastric epithelial cells. Carcinogenesis 21, 1111–1115 (2000). 10836997

[b24] Xu W., Hsu F. F., Baykal E., Huang J. & Zhang K. Sterol biosynthesis is required for heat resistance but not extracellular survival in leishmania. PLoS Pathog. 10, e1004427, 10.1371/journal.ppat.1004427 (2014). 25340392 PMC4207814

[b25] Wriessnegger T. & Pichler H. Yeast metabolic engineering--targeting sterol metabolism and terpenoid formation. Prog. Lipid Res. 52, 277–293, 10.1016/j.plipres.2013.03.001 (2013). 23567752

[b26] Goluszko P. & Nowicki B. Membrane cholesterol: a crucial molecule affecting interactions of microbial pathogens with mammalian cells. Infect. Immun. 73, 7791–7796, 10.1128/IAI.73.12.7791-7796.2005 (2005). 16299268 PMC1307024

[b27] Hamilton J. A. Colony-stimulating factors in inflammation and autoimmunity. Nat. Rev. Immunol. 8, 533–544, 10.1038/nri2356 (2008). 18551128

[b28] Salanga C. L. *et al.* Multiple glycosaminoglycan-binding epitopes of monocyte chemoattractant protein-3/CCL7 enable it to function as a non-oligomerizing chemokine. J. Biol. Chem. 289, 14896–14912, 10.1074/jbc.M114.547737 (2014). 24727473 PMC4031540

[b29] De Filippo K. *et al.* Mast cell and macrophage chemokines CXCL1/CXCL2 control the early stage of neutrophil recruitment during tissue inflammation. Blood 121, 4930–4937, 10.1182/blood-2013-02-486217 (2013). 23645836

[b30] Chen J., Zhao M., Rao R., Inoue H. & Hao C. M. C/EBP{beta} and its binding element are required for NF{kappa}B-induced COX2 expression following hypertonic stress. J. Biol. Chem. 280, 16354–16359, 10.1074/jbc.M411134200 (2005). 15713664

[b31] Macintire K. *et al.* PAPPA2 is increased in severe early onset pre-eclampsia and upregulated with hypoxia. Reprod. Fert. Dev. 26, 351–357, 10.1071/RD12384 (2014). 23484525

[b32] Sok J. *et al.* CHOP-Dependent stress-inducible expression of a novel form of carbonic anhydrase VI. Mol. Cell. Biol. 19, 495–504 (1999). 9858573 10.1128/mcb.19.1.495 PMC83907

[b33] Li Y., He W., Liu T. & Zhang Q. A new cyclo-oxygenase-2 gene variant in the Han Chinese population is associated with an increased risk of gastric carcinoma. Mol. Diagn. Ther. 14, 351–355, 10.2165/11586400-000000000-00000 (2010). 21275453

[b34] Warszawska J. M. *et al.* Lipocalin 2 deactivates macrophages and worsens pneumococcal pneumonia outcomes. J. Clin. Invest. 123, 3363–3372, 10.1172/JCI67911 (2013). 23863624 PMC3726165

[b35] Marks P. W., Arai M., Bandura J. L. & Kwiatkowski D. J. Advillin (p92): a new member of the gelsolin/villin family of actin regulatory proteins. J. Cell Sci. 111, 2129–2136 (1998). 9664034 10.1242/jcs.111.15.2129

[b36] Niess J. H. *et al.* CX3CR1-mediated dendritic cell access to the intestinal lumen and bacterial clearance. Science 307, 254–258, 10.1126/science.1102901 (2005). 15653504

[b37] Fu J., Bian M., Jiang Q. & Zhang C. Roles of Aurora kinases in mitosis and tumorigenesis. Mol. Cancer Res. 5, 1–10, 10.1158/1541-7786.MCR-06-0208 (2007). 17259342

[b38] Song J., Salek-Ardakani S., So T. & Croft M. The kinases aurora B and mTOR regulate the G1-S cell cycle progression of T lymphocytes. Nat. Immunol. 8, 64–73, 10.1038/ni1413 (2007). 17128276

[b39] Papetti M. & Augenlicht L. H. MYBL2, a link between proliferation and differentiation in maturing colon epithelial cells. J. Cell. Physiol. 226, 785–791, 10.1002/jcp.22399 (2011). 20857481 PMC3012743

[b40] Brachner A. *et al.* The endonuclease Ankle1 requires its LEM and GIY-YIG motifs for DNA cleavage *in vivo*. J. Cell Sci. 125, 1048–1057, 10.1242/jcs.098392 (2012). 22399800 PMC4335191

[b41] Kuehnle K. *et al.* Prosurvival effect of DHCR24/Seladin-1 in acute and chronic responses to oxidative stress. Mol. Cell. Biol. 28, 539–550, 10.1128/MCB.00584-07 (2008). 17984220 PMC2223428

[b42] Crameri A. *et al.* The role of seladin-1/DHCR24 in cholesterol biosynthesis, APP processing and Abeta generation *in vivo*. EMBO J. 25, 432–443, 10.1038/sj.emboj.7600938 (2006). 16407971 PMC1383521

[b43] Le P. *et al.* A high-fat diet induces bone loss in mice lacking the Alox5 gene. Endocrinology 153, 6–16, 10.1210/en.2011-0082 (2012). 22128029 PMC3249675

[b44] Shen G. Q. *et al.* An LRP8 variant is associated with familial and premature coronary artery disease and myocardial infarction. Am. J. Hum. Genet. 81, 780–791, 10.1086/521581 (2007). 17847002 PMC2227927

[b45] Zhang J. *et al.* LRP8 mediates Wnt/beta-catenin signaling and controls osteoblast differentiation. J. Bone Miner. Res. 27, 2065–2074, 10.1002/jbmr.1661 (2012). 22589174

[b46] Assimes T. L. *et al.* Common polymorphisms of ALOX5 and ALOX5AP and risk of coronary artery disease. Hum. Genet. 123, 399–408, 10.1007/s00439-008-0489-5 (2008). 18369664 PMC4023692

[b47] Zhuang Y. *et al.* Helicobacter pylori-infected macrophages induce Th17 cell differentiation. Immunobiology 216, 200–207, 10.1016/j.imbio.2010.05.005 (2011). 21112468

[b48] Sharpe A. H., Wherry E. J., Ahmed R. & Freeman G. J. The function of programmed cell death 1 and its ligands in regulating autoimmunity and infection. Nat. Immunol. 8, 239–245, 10.1038/ni1443 (2007). 17304234

[b49] Adachi T., Wakabayashi C., Nakayama T., Yakura H. & Tsubata T. CD72 negatively regulates signaling through the antigen receptor of B cells. J. Immunol. 164, 1223–1229 (2000). 10640734 10.4049/jimmunol.164.3.1223

[b50] Yamazaki T., Nagumo H., Hayashi T., Sugane K. & Agematsu K. CD72-mediated suppression of human naive B cell differentiation by down-regulating X-box binding protein 1. Eur. J. Immunol. 35, 2325–2334, 10.1002/eji.200425639 (2005). 16047337

[b51] Bouloc A., Bagot M., Delaire S., Bensussan A. & Boumsell L. Triggering CD101 molecule on human cutaneous dendritic cells inhibits T cell proliferation via IL-10 production. Eur. J. Immunol. 30, 3132–3139, 10.1002/1521-4141(200011)30:11<3132::AID-IMMU3132>3.0.CO;2-E (2000). 11093127

[b52] Soares L. R., Tsavaler L., Rivas A. & Engleman E. G. V7 (CD101) ligation inhibits TCR/CD3-induced IL-2 production by blocking Ca2+ flux and nuclear factor of activated T cell nuclear translocation. J. Immunol. 161, 209–217 (1998). 9647226

[b53] Ward Y. *et al.* LPA receptor heterodimerizes with CD97 to amplify LPA-initiated RHO-dependent signaling and invasion in prostate cancer cells. Cancer Res. 71, 7301–7311, 10.1158/0008-5472.CAN-11-2381 (2011). 21978933 PMC6697138

[b54] Nishiyama T., Sykora M. M., Huis in ‘t Veld P. J., Mechtler K. & Peters J. M. Aurora B and Cdk1 mediate Wapl activation and release of acetylated cohesin from chromosomes by phosphorylating Sororin. Proc. Natl. Acad. Sci. USA 110, 13404–13409, 10.1073/pnas.1305020110 (2013). 23901111 PMC3746921

[b55] Amieva M. R. *et al.* Disruption of the epithelial apical-junctional complex by Helicobacter pylori CagA. Science 300, 1430–1434, 10.1126/science.1081919 (2003). 12775840 PMC3369828

[b56] Telford J. L. *et al.* Gene structure of the Helicobacter pylori cytotoxin and evidence of its key role in gastric disease. J. Exp. Med. 179, 1653–1658 (1994). 8163943 10.1084/jem.179.5.1653 PMC2191472

[b57] Lee A. *et al.* A standardized mouse model of Helicobacter pylori infection: introducing the Sydney strain. Gastroenterology 112, 1386–1397 (1997). 9098027 10.1016/s0016-5085(97)70155-0

[b58] Crabtree J. E., Ferrero R. L. & Kusters J. G. The mouse colonizing Helicobacter pylori strain SS1 may lack a functional cag pathogenicity island. Helicobacter 7, 139–140; author reply 140-131 (2002). 11966874 10.1046/j.1083-4389.2002.00071.x

[b59] Alm R. A. *et al.* Genomic-sequence comparison of two unrelated isolates of the human gastric pathogen Helicobacter pylori. Nature 397, 176–180, 10.1038/16495 (1999). 9923682

[b60] Khosravi Y. *et al.* Comparing the genomes of Helicobacter pylori clinical strain UM032 and Mice-adapted derivatives. Gut pathog. 5, 25, 10.1186/1757-4749-5-25 (2013). 23957912 PMC3751790

[b61] Ding S. Z., Smith M. F. Jr. & Goldberg J. B. Helicobacter pylori and mitogen-activated protein kinases regulate the cell cycle, proliferation and apoptosis in gastric epithelial cells. J. Gastroenterol. Hepatol. 23, e67–78, 10.1111/j.1440-1746.2007.04912.x (2008). 18702686

[b62] Scotti C. *et al.* Cell-cycle inhibition by Helicobacter pylori L-asparaginase. PloS one 5, e13892, 10.1371/journal.pone.0013892 (2010). 21085483 PMC2976697

[b63] Johnson D. G. & Walker C. L. Cyclins and cell cycle checkpoints. Annu. Rev. Pharmacol. Toxicol. 39, 295–312, 10.1146/annurev.pharmtox.39.1.295 (1999). 10331086

[b64] Lim S. & Kaldis P. Cdks, cyclins and CKIs: roles beyond cell cycle regulation. Development 140, 3079–3093, 10.1242/dev.091744 (2013). 23861057

[b65] Lei M. The MCM complex: its role in DNA replication and implications for cancer therapy. Curr. Cancer Drug Targets 5, 365–380 (2005). 16101384 10.2174/1568009054629654

[b66] Tye B. K. MCM proteins in DNA replication. Annu. Rev. Biochem. 68, 649–686, 10.1146/annurev.biochem.68.1.649 (1999). 10872463

[b67] Bambara R. A. & Jessee C. B. Properties of DNA polymerases delta and epsilon, and their roles in eukaryotic DNA replication. Biochim. Biophys. Acta 1088, 11–24 (1991). 1846563 10.1016/0167-4781(91)90147-e

[b68] Hubscher U., Maga G. & Spadari S. Eukaryotic DNA polymerases. Annu. Rev. Biochem. 71, 133–163, 10.1146/annurev.biochem.71.090501.150041 (2002). 12045093

[b69] Schmidt S. L., Pautz A. L. & Burgers P. M. ATP utilization by yeast replication factor C. IV. RFC ATP-binding mutants show defects in DNA replication, DNA repair, and checkpoint regulation. J. Biol. Chem. 276, 34792–34800 (2001). 11549622 10.1074/jbc.m011671200

[b70] Andrews P. D., Knatko E., Moore W. J. & Swedlow J. R. Mitotic mechanics: the auroras come into view. Curr. Opin. Cell Biol. 15, 672–683 (2003). 14644191 10.1016/j.ceb.2003.10.013

[b71] Slattery S. D., Moore R. V., Brinkley B. R. & Hall R. M. Aurora-C and Aurora-B share phosphorylation and regulation of CENP-A and Borealin during mitosis. Cell cycle 7, 787–795 (2008). 18239465 10.4161/cc.7.6.5563

[b72] Zeitlin S. G., Shelby R. D. & Sullivan K. F. CENP-A is phosphorylated by Aurora B kinase and plays an unexpected role in completion of cytokinesis. J. Cell Biol. 155, 1147–1157, 10.1083/jcb.200108125 (2001). 11756469 PMC2199334

[b73] Emre D., Terracol R., Poncet A., Rahmani Z. & Karess R. E. A mitotic role for Mad1 beyond the spindle checkpoint. J. Cell Sci. 124, 1664–1671, 10.1242/jcs.081216 (2011). 21511728

[b74] Fava L. L., Kaulich M., Nigg E. A. & Santamaria A. Probing the *in vivo* function of Mad1:C-Mad2 in the spindle assembly checkpoint. EMBO J. 30, 3322–3336, 10.1038/emboj.2011.239 (2011). 21772247 PMC3160659

[b75] Jensen S., Segal M., Clarke D. J. & Reed S. I. A novel role of the budding yeast separin Esp1 in anaphase spindle elongation: evidence that proper spindle association of Esp1 is regulated by Pds1. J. Cell Biol. 152, 27–40 (2001). 11149918 10.1083/jcb.152.1.27 PMC2193664

[b76] Munari F. *et al.* Cytokine BAFF released by Helicobacter pylori-infected macrophages triggers the Th17 response in human chronic gastritis. J. Immunol. 193, 5584–5594, 10.4049/jimmunol.1302865 (2014). 25339679

[b77] Baldari C. T., Lanzavecchia A. & Telford J. L. Immune subversion by Helicobacter pylori. Trends Immunol. 26, 199–207, 10.1016/j.it.2005.01.007 (2005). 15797510

[b78] Boncristiano M. *et al.* The Helicobacter pylori vacuolating toxin inhibits T cell activation by two independent mechanisms. J. Exp. Med. 198, 1887–1897, 10.1084/jem.20030621 (2003). 14676300 PMC2194151

[b79] Molinari M. *et al.* Selective inhibition of Ii-dependent antigen presentation by Helicobacter pylori toxin VacA. J. Exp. Med. 187, 135–140 (1998). 9419220 10.1084/jem.187.1.135 PMC2199184

[b80] Weischenfeldt J. & Porse B. Bone Marrow-Derived Macrophages (BMM): Isolation and Applications. Cold Spring Harb. Protoc. 2008, pdb prot5080, 10.1101/pdb.prot5080 (2008). 21356739

[b81] Lacey D. C. *et al.* Defining GM-CSF- and macrophage-CSF-dependent macrophage responses by *in vitro* models. J. Immunol. 188, 5752–5765, 10.4049/jimmunol.1103426 (2012). 22547697

[b82] Wong W. F., Kurokawa M., Satake M. & Kohu K. Down-regulation of Runx1 expression by TCR signal involves an autoregulatory mechanism and contributes to IL-2 production. J. Biol. Chem. 286, 11110–11118, 10.1074/jbc.M110.166694 (2011). 21292764 PMC3064165

[b83] Kanehisa M. & Goto S. KEGG: kyoto encyclopedia of genes and genomes. Nucleic Acids Res. 28, 27–30 (2000). 10592173 10.1093/nar/28.1.27 PMC102409

[b84] Kanehisa M. *et al.* Data, information, knowledge and principle: back to metabolism in KEGG. Nucleic Acids Res. 42, D199–205, 10.1093/nar/gkt1076 (2014). 24214961 PMC3965122

[b85] Saeed A. I. *et al.* TM4: a free, open-source system for microarray data management and analysis. BioTechniques 34, 374–378 (2003). 12613259 10.2144/03342mt01

[b86] Looi C. Y. *et al.* Induction of apoptosis in human breast cancer cells via caspase pathway by vernodalin isolated from Centratherum anthelminticum (L.) seeds. PloS one 8, e56643, 10.1371/journal.pone.0056643 (2013). 23437193 PMC3577860

[b87] Wong W. F. *et al.* T-cell receptor signaling induces proximal Runx1 transactivation via a calcineurin-NFAT pathway. Eur. J. Immunol. 44, 894–904, 10.1002/eji.201343496 (2014). 24310293

