# Supplementary material for: Suppression of cell division-associated genes by Helicobacter pylori attenuates proliferation of RAW264.7 monocytic macrophage cells
Source: Sci Rep. 2015 Jun 16;5:11046. doi: 10.1038/srep11046 (PMC4468580; doi:10.1038/srep11046)
Supplement: Supplementary Information [file srep11046-s1.pdf]

Supplementary Figures and Tables

Suppression of cell division-associated genes by *Helicobacter pylori* attenuates proliferation of RAW264.7 monocytic macrophage cells

Grace Min Yi Tan[1], Chung Yeng Looi[2], Keith Conrad Fernandez[1], Jamuna Vadivelu[1], Mun Fai Loke[1*] & Won Fen Wong[1,3*]

[1]Department of Medical Microbiology, and [2]Department of Pharmacology, Faculty of Medicine, University of Malaya, 50603 Kuala Lumpur, Malaysia, [3]Tropical Infectious Disease Research and Education Center, University of Malaya, 50603 Kuala Lumpur, Malaysia.

Supplementary Figure S1

Supplementary Figure S2

Supplementary Figure S3

Supplementary Figure S4

Supplementary Table S1

Supplementary Table S2

Figure S1. Apoptosis assay. RAW264.7 cells were infected with the indicated MOIs of *H. pylori* for 24 h. Cells were stained with annexin V and propidium iodide from apoptosis kit (BD Biosciences), and analyzed in a flow cytometer.

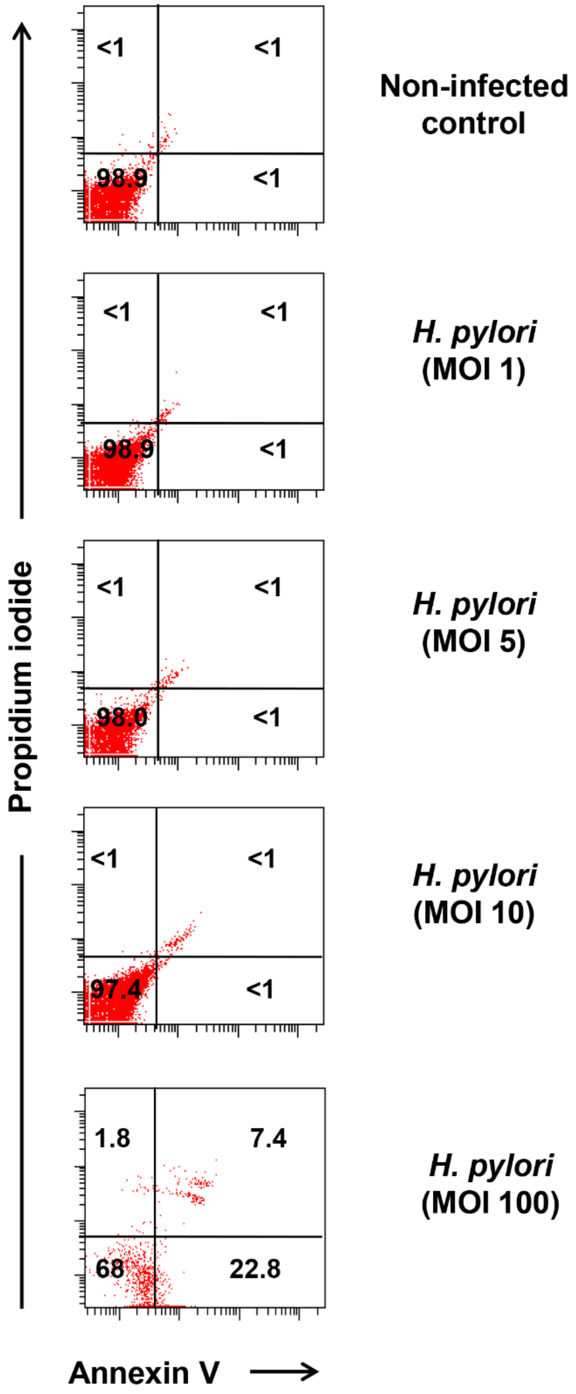

Figure S2. Q-RT-PCR analysis. RNA were extracted from non-infected (-) or *H. pylori* (MOI 10, 24 h)-infected RAW264.7 cells. *P<0.05, **P<0.01, ***P<0.001.

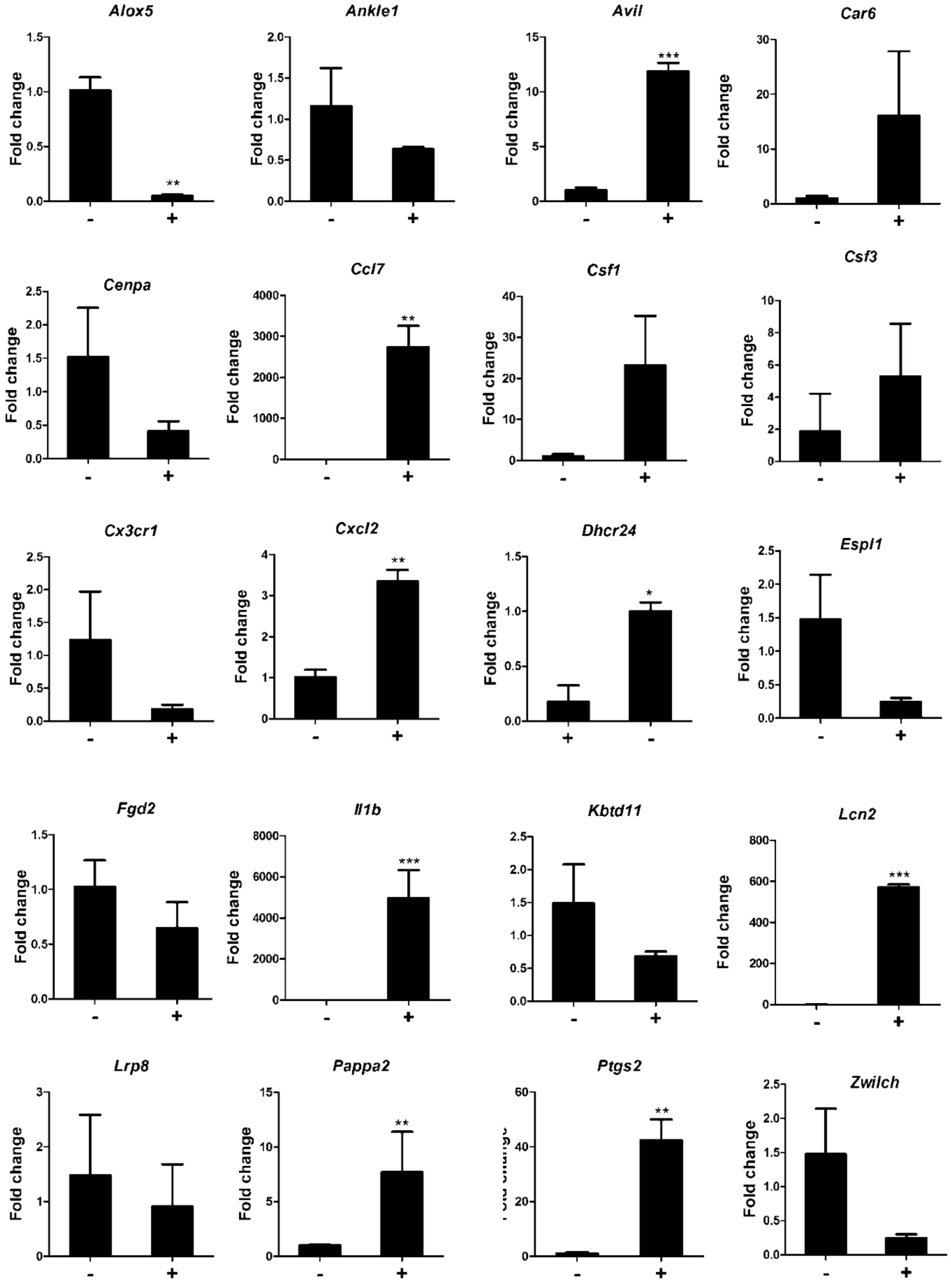

Figure S3. Effect of different strains of *H. pylori* infection on cell cycle of RAW264.7 cells. RAW264.7 cells were uninfected or infected with SS1, J99 or 298 strains at MOI 100 for 24 h. Cells were harvested at 24 hpi, fixed and stained with propidium iodide (PI) to detect DNA contents. Numbers represent the percentages of cells at G0/G1, S or G2/M phases.

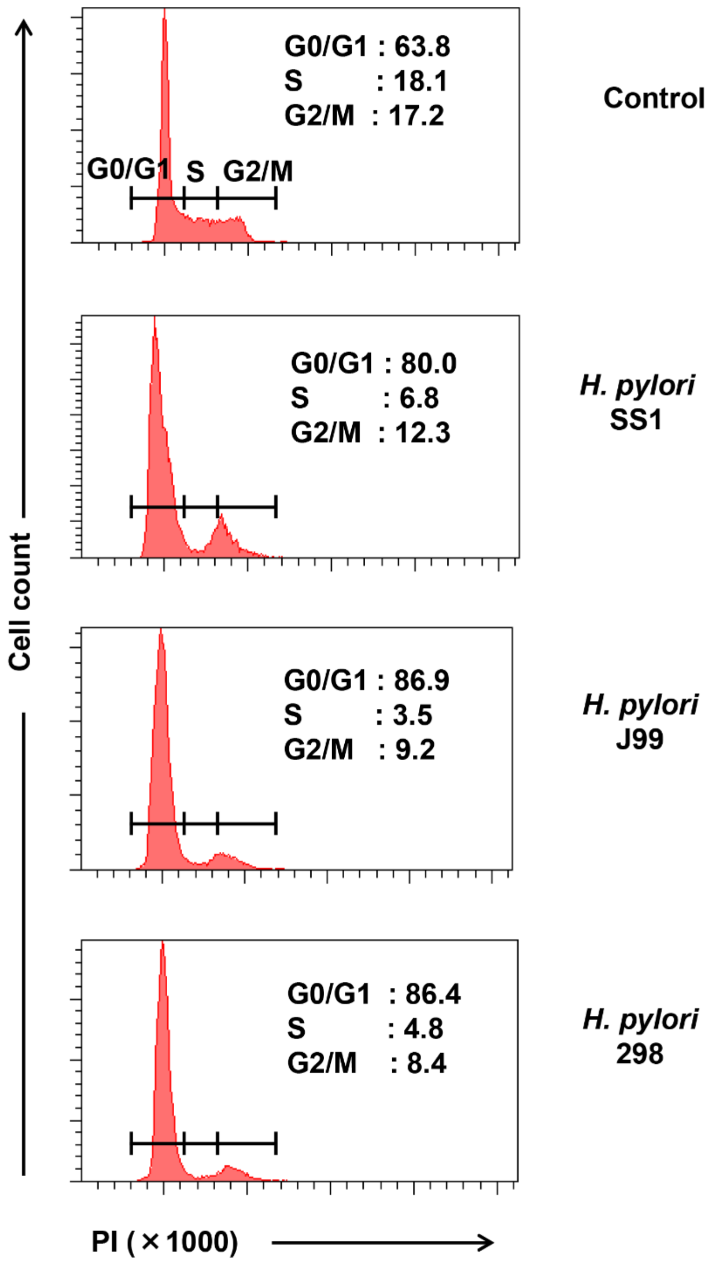

Figure S4. Full length gel images correspond to cropped images in Figure 6b. Rectangles (dotted lines) over the gels indicate where the images for the Figure 5b were cropped.

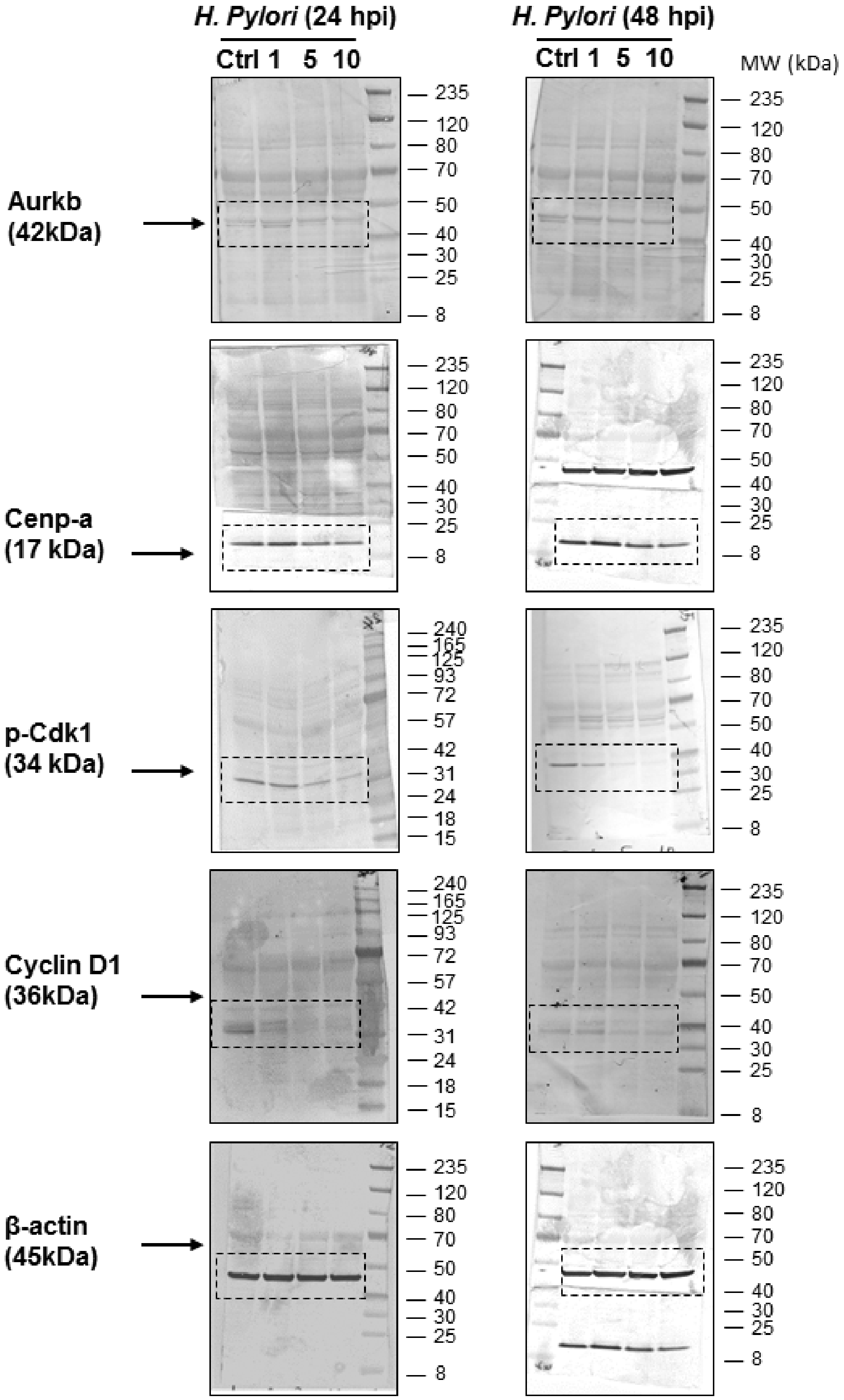

Table S1. KEGG pathway analysis. List of top 5 pathways for genes up-regulated by *H. pylori* infection in RAW264.7 cells. Pathways were filtered with P<0.05 and was presented according to number of genes that were up-regulated or down-regulated in each pathway. The statistical significances were also analyzed with Bonferroni, Benjamini and FDR tests.

Term	Count	FE	Up-regulated Genes	Statistic
mmu04142: Lysosome	33	3.94	*NAGLU, MFSD8, ATP6AP1, AP3S1, CTSA, ACP2, ATP6V0B, ATP6V0C, SLC11A2, AP1S3, SLC11A1, AP3M2, TPP1, NAGA, ATP6V0D1, ATP6V0D2, IDUA, TCIRG1, CLN3, CTSZ, PSAP, ATP6V1H, CD63, GNS, CTSL, NPC1, SLC17A5, LAMP2, GAA, ATP6V0A1, CTSD, GGA1, CLN5*	P: 1.20×10^{-11} Bonferroni: 2.05×10^{-9} Benjamini:2.05×10^{-9} FDR: 1.47×10^{-8}
mmu04060: Cytokine-cytokine receptor interaction	31	1.80	*CSF3, CSF2, CCL3, CCL2, TNF, CSF2RB2, CCR1, CSF1, CXCL2, CCL9, TNFSF12, IL7R, CCL4, CCL7, IL11, TNFRSF1B, IL23A, IL10RB, CSF2RB, IL1B, IL2RG, FAS, IL1A, LTA, IL11RA1, CD40, OSM, VEGFC, ACVR2A, CCR1L1, ACVR1*	P: 0.0016 Bonferroni: 0.2404 Benjamini:0.0875 FDR: 1.94
mmu00190: Oxidative phosphorylatio n	16	1.75	*TCIRG1, NDUFA3, ATP6AP1, ATP6V1H, ATP6V1G1, ATP6V1D, ATP6V0B, ATP6V1F, ATP6V0C, ATP6V1C1, ATP6V1A, ATP6V1E1, COX6A2, ATP6V0A1, ATP6V0D1, ATP6V0D2*	P: 0.0373 Bonferroni: 0.9985 Benjamini:0.3032 FDR: 37.15
mmu04512: ECM-receptor interaction	13	2.23	*ITGB4, ITGB5, ITGB3, COL5A3, VWF, CD47, CD36, CD44, ITGA6, ITGB7, ITGAV, ITGA7, SPP1*	P: 0.0122 Bonferroni: 0.8777 Benjamini:0.2310 FDR: 13.93
mmu05414: Dilated cardiomyopath y	13	2.01	*TNF, TNNC1, MYBPC3, ITGB4, ITGB5, ITGB3, TPM1, PRKX, ITGA6, ITGB7, ITGAV, ITGA7, SGCB*	P: 0.0259 Bonferroni: 0.9889 Benjamini:0.3129 FDR: 27.50
**Term**	**Count**	**FE**	**Down-regulated Genes**	**Statistic**
mmu03030: DNA replication	27	-8.27	*POLA1, POLA2, RPA1, PRIM1, RPA2, MCM7, POLE2, PRIM2, FEN1, LIG1, POLE, MCM2, RNASEH2A, MCM3, MCM4, RNASEH2C, MCM5, MCM6, RFC5, DNA2, RFC3, RFC4, RFC1, RFC2, POLD1, POLD2, PCNA*	P: 2.68×10^{-20} Bonferroni: 4.78×10^{-18} Benjamini: 2.39×10^{-18} FDR: 3.30×10^{-17}
mmu03430: Mismatch repair	15	-7.31	*EXO1, MSH6, MSH2, LIG1, RFC5, RPA1, RPA2, RFC3, RFC4, RFC1, RFC2, POLD1, POLD2, PCNA, PMS2*	P: 4.93×10^{-10} Bonferroni: 8.77×10^{-8} Benjamini: 2.92×10^{-8} FDR: 6.06×10^{-7}
mmu03420: Nucleotide	14	-3.49	*LIG1, POLE, RFC5, RPA1, RPA2, RFC3, RFC4, POLE2, RFC1, RFC2, POLD1,*	P: 9.02×10^{-5} Bonferroni: 0.01594

excision repair			*POLD2, DDB2, PCNA*	Benjamini: 0.00161<br>FDR: 0.11089
mmu00100: Steroid biosynthesis	11	-6.93	*TM7SF2, CYP51, EBP, SQLE, DHCR7, LSS, HSD17B7, SC4MOL, NSDHL, DHCR24, FDFT1*	P: 4.78×10^{-7}<br>Bonferroni: 8.52×10^{-5}<br>Benjamini: 2.13×10^{-5}<br>FDR: 5.88×10^{-4}
mmu00900: Terpenoid backbone biosynthesis	10	-7.66	*MVD, HMGCR, FDPS, HMGCS1, MVK, PMVK, ACAT2, IDI1, ACAT1, PDSS1*	P: 6.41×10^{-7}<br>Bonferroni: 1.14×10^{-4}<br>Benjamini: 2.28×10^{-5}<br>FDR: 7.89×10^{-4}

Table S2. Primer sequences for qRT-PCR analysis. List of the forward and reverse primer sequences (5’-3’) of the genes selected for qRT-PCR analysis.

Gene Symbol	Forward primer	Reverse primer
Alox5	ACAGGAAGTACAGGAAGGGAAC	GGGTTACTCTCTCCAGGGGT
Ankle1	CTCTTCCCTCACACTGGCTG	AGACCTGTTTCCTTGGCTGG
Aurkb	CTGGAGAATGGCTCAGAAGG	ACAGTGATGGGGGAGAAATG
Avil	CGCTAATGACAAAAGGCTGC	CCTCCGCTCCTATCCACAAG
Car6	CGTGGGAGAAAGTCAGTGGT	GCCTAGCTGTGTTAGCCGTC
Ccl7	TCTGTGCCTGCTGCTCATAG	CATTCCTTAGGCGTGACCAT
Ccnb1	CTCCCTTTCATCCACAGGAA	TTGCAGTCTGCCTTCTCTCA
Ccnb2	CAGTTCCCAAATCCGAGAAA	GAACAAGTATGCCAGCAGCA
Ccne1	ACTTTCTGCAGCGTCATCCT	CTATGTCAACGACACGGGTG
Ccne2	GACTGGATGGTGCCTTTTGT	GATGCACAAAATCCTGGGTT
Cdk1	CCCACCTACGGACAGTGTTT	CGTATCGCTGTGCTGCTTAG
Cdk2	TAAGTGCTGTGGGGAACACA	TCACCCTTTCTTCCAGGATG
Cenpa	CCAGCCACTGAGAGTCACAA	TTGGGAACTGTAGGTGAGGGC
Csf1	CCCTGGCTACTGGGTCAATA	AAAGCTCAGAGGGAGCACTG
Csf3	CTCAACTTTCTGCCCAGAGG	GCTCTATCGGGTATTTCCCC
Cx3cr1	GGAGACTGGAGCCAACAGAG	CAGGACACAGCCAGACAAGA
Cxcl2	GCCTGGATCGTACCTGATGT	GGAAGGAGTGTGCATGTTCA
Dhcr24	GACATCCAGAAACAGGTCCG	ATAGACACCAAGGGCTCCAC
Espl1	CTCTTGAACCTTGGCTGTCC	CATCATTCTGCTCATGGGAA
Fgd2	TCTGCCTGACCTGCTACA	GTGAGCCTTTGTGTCCTGG
Il1b	TGCCACCTTTTGACAGTGATG	ACGGGAAAGACACAGGTAGC
Kbtd11	ACAGGTGCGTGGAAGGT	CTATGGGCGAGTGTCTGGAA
Lcn2	AATGTCACCTCCATCCTGGTC	GCTCCTTGGTTCTTCCATACAG
Lrp8	CACACGGATTGGTTTCACTG	CAGAAGGAATTCAGTCCCCA
Mad1l1	TAGACCGGTGCGAGAGAGAT	AAAACAGCAGAGTCAGGGGA

Pappa2	GAAGTGCTGGCTGAGATTCC	GCTCTTTAATCCTGGGAGGG
Ptgs2	GCAGGAAGTCTTTGGTCTGG	TCCTCCTGGAACATGGACTC
Zwilch	TTGCCCCATCACAAACTACA	TTTAATTCCAGCACTTGGGG
